# Limitation of O_3_ Dry Deposition by Elevated-Source NO Emissions and Surface Dryness in Urban Environments

**DOI:** 10.1021/acs.est.6c05093

**Published:** 2026-09-22

**Authors:** Daniel Choi, Yibo Huangfu, Erick Fredj, Qian Li, Bin Yuan, Xiaoxiao Zhang, Xianjun He, Huizhi Liu, Eran Tas

**Affiliations:** † The Robert H. Smith Faculty of Agriculture, Food and Environment, Department of Soil and Water Sciences, The Hebrew University of Jerusalem, Rehovot 7610001, Israel; ‡ College of Environment and Climate, Institute for Environmental and Climate Research, Guangdong-Hongkong-Macau Joint Laboratory of Collaborative Innovation for Environmental Quality, Jinan University, Guangzhou 511443, China; § Department of Computer Science, 42730 Jerusalem College of Technology, Jerusalem 91160, Israel; ∥ School of Ecology and Environment, Hainan University, Haikou, Hainan Province 570228, China; ⊥ Department of Atmospheric Sciences, Yunnan University, Kunming 650500, China

**Keywords:** O_3_, dry deposition, RH, NO, NO_*x*_, urban air pollution, surface uptake

## Abstract

Tropospheric ozone (O_3_) is a major air pollutant that adversely affects human health, climate and ecosystems. However, its removal in urban environments remains poorly represented in air-quality and climate models, largely due to limited flux-based observations of urban O_3_ deposition. Using eddy-covariance flux measurements from a meteorological tower in Beijing, we show—for the first time to our knowledge—that surface uptake plays a central role in O_3_ removal in urban environments but is strongly limited under dry conditions by evaporation and by higher-above-measurement nitrogen monoxide (NO) concentrations (HAMNC), inferred from downward NO fluxes and likely linked to elevated-source NO emissions. These limitations led to significantly more frequent positive ozone-flux events under low relative humidity (RH < 30%) and downward NO flux, reducing downward O_3_ flux by ∼5% and ∼3%, respectively. We provide explicit functional relationships describing the response of downward O_3_ velocity (Vdw,O_3_) to both near-surface dryness (RH) and HAMNC, accounting for processes missed by routine ground-based measurements that can lead to misinterpretation of O_3_ removal in the urban environment and beyond. Incorporating the RH-dependent limitation of Vdw,O_3_ into air-quality and climate models is critical, given that ∼40% of the global land surface is arid or semiarid.

## Graphical abstract

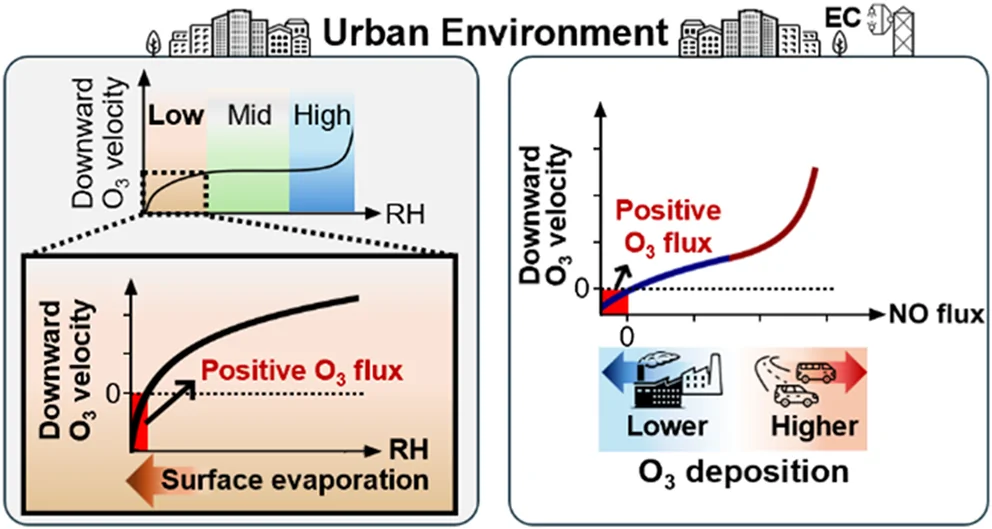

## 1 Introduction

Tropospheric ozone (O_3_) is a major air pollutant associated with approximately one million premature deaths globally^1^; it contributes significantly to global warming^2–4^ and poses a threat to food security by reducing crop yields.^5,6^ Global urban O_3_ levels have increased on average by 0.31 ppb year^–1^ since the 1990s,^7^ highlighting the need to understand the processes controlling O_3_ removal, particularly in urban areas.

Dry deposition accounts for about 20–25% of tropospheric O_3_ removal,^8,9^ but reliable simulations of O_3_ mixing ratios by climate and air-quality models are limited by insufficiently accurate representation of O_3_ dry-deposition rates (Vd,O_3_).^10–14^ While O_3_ dry deposition has been studied mainly in vegetated environments focusing on both stomatal and nonstomatal pathways, its characterization in urban areas remains poor due to the scarcity of direct urban O_3_-flux measurements.^12–15^

Recently, Karl et al.^16^ showed that chemical removal of O_3_ by NO can dominate the observed downward O_3_ velocity (Vdw,O_3_) in the urban environment, implying that the contribution of dry deposition to Vdw,O_3_ in urban areas can be relatively minor. Subsequent studies by Zhang et al.^17^ and Choi et al.^18^ confirmed the important role of NO in O_3_ removal, while Choi et al.^18^ also highlighted the contribution of surface uptake to Vdw,O_3_, as indicated by a strong correlation between relative humidity (RH) and Vdw,O_3_.

They further showed that both low RH and higher-above-measurement NO concentrations (HAMNC), likely associated with NO emissions from elevated sources (i.e., sources that may lead to HAMNC, such as power-plant and industrial stacks; Section S2.2 of the Supporting Information), drive positive ozone-flux events (POFEs), which occurred during approximately 9% of the observations. HAMNC describes conditions in which NO concentrations above the eddy covariance (EC) measurement height exceed those below it, resulting in stronger O_3_ titration above than below the EC measurement height, thereby producing a POFE. Under low-RH conditions, POFEs likely reflect very high surface resistance that suppresses surface O_3_ uptake.^18,19^

The occurrence of these POFEs suggests that near-surface dryness and HAMNC may play a substantial role in limiting O_3_ dry deposition. Therefore, a better characterization of how RH and HAMNC affect Vdw,O_3_ is needed. Improved characterization of limitations on surface O_3_ uptake under low-RH conditions is critical, as this process is not represented in current air-quality and climate models,^14^ although semiarid and arid regions cover about 40% of Earth’s land surface.^20^ In addition, reductions in Vdw,O_3_ due to HAMNC cannot be captured by routine ground-based measurements, even though these reductions may significantly affect the interpretation of the factors controlling O_3_ dry deposition.

Because factors that enhance or inhibit surface O_3_ uptake, including enhancement by near-ground NO emissions and inhibition by HAMNC and surface dryness, can act simultaneously and mask each other, comprehensive measurements are needed to disentangle their contributions to O_3_ removal. We hypothesize that an urban environment characterized by a wide range of RHs, together with strong, temporally variable NO emissions from road traffic and from elevated sources that can lead to HAMNC, provides suitable conditions to isolate and characterize the effects of low RH and HAMNC on Vdw,O_3_, with relevance for O_3_ modeling and assessment from urban to global scales. Accordingly, we used summer flux measurements in urban Beijing to isolate and characterize these effects, with POFEs serving as diagnostic indicators of their impact on O_3_ deposition.

## 2 Materials and Methods

### 2.1 Measurement Site, Instrumentation, and Flux Calculation

The measurements were conducted between 2 May and 25 June 2023 from the 325 m meteorological tower of the Institute of Atmospheric Physics (IAP), Chinese Academy of Sciences, Beijing (39°58′N, 116°22′E; ∼60 m a.s.l.). The IAP tower is a lattice structure that reduces tower-body flow interference. The surrounding area includes a small park to the west (∼0.3 km^2^), major roads to the east and north, and dense residential areas to the south and north with low vegetation cover.^17,18^ The fluxes of O_3_, NO_*x*_, and VOCs were quantified using the EC technique.^21–23^ The core of the data set corresponds to an elevation of 102 m above ground level. A 3D sonic anemometer (IRGASON 3D sonic anemometer; Campbell Scientific, USA) was mounted at this elevation, more than twice the mean height (∼45 m) of the tallest residential buildings within the flux footprint,^24^ ensuring that the instrument was positioned above the roughness sublayer and within the inertial sublayer.^17,25^ This setup allowed quantification of the turbulent three-dimensional wind components at 10 Hz.

Sampling inlets were placed close to the sonic anemometer to support measurements of O_3_, VOCs, and NO_*x*_ mixing ratios. The turbulent fluctuations of O_3_ were quantified using a 10 Hz O_3_ sensor (FOS V2.0.1 chemiluminescence sensor; Sextant Technology, New Zealand).^17^ The sensor signal was calibrated to yield O_3_ mixing ratios by comparing 30 min averaged FOS measurements with simultaneous measurements from a 1 Hz UV photometric O_3_ analyzer (Model 205; 2B Technologies, USA).^17^

A dual-channel chemiluminescence instrument was used for the quantification of NO and NO_2_ at 1 Hz.^17^ VOC mixing ratios were quantified at 10 Hz using a Vocus Proton-Transfer-Reaction Time-of-Flight Mass Spectrometer (Vocus-PTR-TOF-MS; Tofwerk AG, Switzerland). The instrumentation for O_3_, NO_*x*_, and VOCs was housed in an air-conditioned laboratory. Air from the 102 m inlets was supplied to the instruments using a high-volume vacuum pump (Gast, Model 1023–101QSG608) operating at an actual flow rate of ∼37 L min^–1^ in the inlet line, pulling air through ∼150 m of 0.9 cm I.D. PFA tubing.^17^

Concurrent with the EC measurements, tower-based profiles of RH, air temperature (*T*), and wind speed (WS) and direction were derived from 15 fixed levels between 8 and 320 m above ground level on the IAP meteorological tower. In the following, subscripts attached to meteorological variables indicate the measurement height (*m*); e.g., *Y_Xm_* denotes variable *Y* measured at *X* meters. Vapor-pressure deficit (VPD) was calculated as the difference between saturation vapor pressure and actual vapor pressure.

Fluxes of O_3_, NO, and NO_2_ (FO_3_, FNO, and FNO_2_, respectively) were calculated using InnFlux software.^26^ A 30 min averaging interval was used for flux calculations. This averaging interval is sufficiently long to capture the contribution of the largest relevant turbulent eddies,^27,28^ while limiting the influence of the lower-frequency variability unrelated to turbulent transport.^29^ The suitability of this averaging interval for the present data set was supported by stationarity and cospectral analyses. A detailed description of the EC processing framework, including the stationarity and cospectral analyses performed for this data set, is provided in Zhang et al.^17^ Daytime conditions were defined as 30 min periods with positive global solar radiation (GSR > 0), and all daytime analyses in this study were based on these 30 min averaged observations. Data processing included double rotation to correct for sonic anemometer tilt before flux calculation.^30^ The quality of the flux data was evaluated using steady-state tests to exclude periods that were not sufficiently stationary (30% threshold),^17^ while tests of developed turbulence conditions were used to ensure that atmospheric turbulence was sufficiently developed for reliable EC flux measurements.^31^ Fluxes were assigned a 10-level quality index (*Q*_class_), and only data with *Q*_class_ ≤ 6 were retained for analysis.^17^ As an additional uncertainty assessment, the random-flux uncertainty associated with the O_3_ flux was calculated for each 30 min period from the high-frequency turbulent fluctuations in vertical wind velocity and O_3_ concentration using the random-shuffle method of Billesbach^32^ (see Section S6).

The flux footprint was evaluated using the 2D parametrization of Kljun et al.,^33^ as applied in Zhang et al.^17^ The resulting 90% footprint covers approximately 70 km^2^ and consists mainly of urban land-use types (residential ∼55%, commercial ∼13%, office ∼5%, administrative ∼7%, educational ∼11%), while parks and green spaces represent ∼16% of the area.^34^ This heterogeneous composition indicates that the measured fluxes are broadly representative of the urban environment in Beijing.^34^

### 2.2 POFE Definition and Classification Criteria

The flux analyses identified POFEs that could be attributed to two distinct event types (see Section 1). A relatively high proportion of these POFEs was associated with dry conditions (typically RH < 30%), whereas many others coincided with a downward FNO, suggesting HAMNC, which in most cases was likely associated with NO emissions from elevated sources. Because positive O_3_ fluxes are atypical in the context of surface-layer O_3_ dry deposition, we performed an additional uncertainty assessment of the positive O_3_ fluxes defining POFEs by comparing their magnitudes with the corresponding random-flux uncertainty estimates (Section S6).

Accordingly, we defined POFE1 as 30 min averaging periods in which mean RH < 30% and the corresponding mean FO_3_ was positive. POFE2 was defined as 30 min averaging periods in which a mean positive FO_3_ was observed together with a downward mean FNO (FNO < 0). In many cases, these POFE2s also coincided with a negative FNO_2_ (FNO_2_ < 0), but FNO_2_ was generally not considered in the definition of POFE2s (see Table 1). FO_3_ > 0, FNO < 0 and FNO_2_ < 0 refer to 30 min averaging periods for which the corresponding mean values satisfied $\overset{®}{{\mathrm{FO}}_{3}}>0$ , $\overset{®}{\mathrm{FNO}}<0$ , or $\overset{®}{{\mathrm{FNO}}_{2}}<0$ , respectively. To exclude the effect of O_3_ mixing ratios (CO_3_) on the measured flux, we calculated the observed downward O_3_ velocity, Vdw,O_3_, as

$$Vdw,O_{3}=-\frac{{\mathrm{FO}}_{3}}{{\mathrm{CO}}_{3}}$$

where FO_3_ is the measured vertical flux of O_3_, and CO_3_ is its measured mixing ratio. By convention, FO_3_ < 0 denotes a downward flux, so Vdw,O_3_ is expressed as a positive value for non-POFEs and as a negative value for POFEs. The occurrence of POFEs as POFE1 (RH < 30%) and POFE2 (FNO < 0) was evaluated using Fisher’s exact test with a significance threshold of *p* < 0.01.

**Table 1:** Summary of Positive Ozone-Flux Events (POFEs)^a^

		POFE occurrence		Condition				Mean mixing ratios		
POFE/Criteria	D/N	Total occurrence	POFEs (FO_3_ > 0)	RH_8m_ < 30%	FNO < 0	FNO ≥ 0	FNO_2_ < 0	NO (ppb)	NO_2_ (ppb)	NO_*x*_ (ppb)
POFE1	Day	46	45 (98%)	45 (98%)	0 (0%)	34 (74%)	0 (0%)	0.94	4.41	4.89
	Night		1 (2%)	1 (2%)	N/A^c^	N/A^c^	N/A^c^	N/A^c^	N/A^c^	N/A^c^
POFE2	Day	29	16 (55%)	1 (3%)	16 (55%)	N/A^d^	11 (38%)	6.88	29.90	36.21
	Night		13 (45%)	0 (0%)	12 (41%)^b^	N/A^d^	10 (34%)	2.02	22.89	24.47
Missing data	Day	10	8 (80%)	0 (0%)	N/A^c^	N/A^c^	0 (0%)	5.75	24.80	30.10
	Night		2 (20%)	0 (0%)	N/A^c^	N/A^c^	0 (0%)	1.19	13.19	13.94
Total	Day	123	93 (76%)	46 (37%)	16 (13%)	58 (47%)	11 (10%)	3.16	14.46	16.97
	Night		30 (24%)	1 (1%)	12 (10%)	14 (11%)	12 (10%)	1.50	17.88	18.94
	Total		123 (100%)	47 (38%)	28 (23%)	72 (59%)	23 (19%)	2.75	15.30	17.45

a Shown are occurrence frequencies (counts) for each condition of relative humidity (RH_8m_), NO flux (FNO) and NO_2_ flux (FNO_2_) direction, along with mean mixing ratios of NO, NO_2_ and NO_*x*_. The table reports type 1-classified POFEs (POFE1), type 2-classified POFEs (POFE2) and POFEs with missing data; POFE1 + POFE2 + missing data + unclassified POFEs (not shown here) yields the total. Values in parentheses are percentages calculated relative to the total occurrence in each row.

b Percentage was calculated excluding a single case that was classified as POFE2 based on a negative NO_2_ flux, for which NO flux data were unavailable.

c No data available.

d Not defined: POFE2 is only applicable when FNO < 0.

## 3 Results and Discussion

VOCs can react with O_3_ and therefore influence O_3_ removal and the observed O_3_ flux. Analysis of the measured data (Section S4) indicated that the investigated VOC fluxes showed only weak associations with Vdw,O_3_, whereas NO fluxes dominated the observed variability in Vdw,O_3_. For this reason, VOCs are not addressed further in the present study, which focuses on the dominant inhibitory effects of HAMNC and surface dryness. A more detailed analysis of the role of VOCs in O_3_ removal based on this data set is provided in Choi et al.^18^

### 3.1 Observed POFEs: Characteristics, Frequency, and Effects

POFEs occurred in approximately 9% of the observations. Among those that could be classified, the events were primarily associated with either low RH (in particular RH < 30%; POFE1) or a negative FNO (POFE2) (see Sections 1 and 2.2), enabling investigation of the effects of low RH and HAMNC on Vdw,O_3_. About 39% of the POFEs could not be classified as either POFE1 or POFE2 because data were missing or the conditions defining POFE1 or POFE2 were not fully met. POFE1 appears to be driven by low RH, which suppresses surface wetness, increases surface resistance to O_3_ uptake, and thereby limits O_3_ deposition. POFEs occurred more frequently at RH < 30%, apparently associated with extremely high surface resistance (see Section 3.3.1). The negative FNO in POFE2 (see analysis in Section S1) is consistent with HAMNC-induced titration, which leads to a reversal of the vertical O_3_ gradient across the EC measurement height and results in a positive O_3_ flux.^35^

Table 1 summarizes the frequency of the observed POFEs, together with the corresponding RH, FNO values and NO and NO_2_ mixing ratios. A quantitative analysis of the NO–NO_2_–O_3_ system for the same data set showed that it was consistent with efficient in situ NO–O_3_ titration and was governed primarily by local photochemistry rather than long-range transport.^17,18^ Our analysis revealed relatively higher NO_*x*_ mixing ratios during POFE2s (Table 1). This is consistent with a contribution from elevated emission sources, which can lead to HAMNC during these events. POFE2s were typically associated with local west-southwesterly (WSW) to northwesterly (NW) wind directions, between 103 and 240 m (see Section S1, Figure S1), suggesting that the NO emissions responsible for the POFE2s predominantly originated from sources located within the WSW-NW sectors relative to the measurement site. Several additional elevated combustion sources are distributed around the IAP tower (Figure S5), and their relative contribution to HAMNC formation was found to depend on wind direction, transport conditions, and the concurrent influence of near-ground traffic emissions, which act to mask the influence of the elevated sources (see Section S2.2).

Using the definitions of POFE1 and POFE2 (Section 2.2), our analysis shows that approximately 39% of the observed POFEs could not be classified as either POFE1 or POFE2. Among these, about 21% (8% of all POFEs) could not be classified as either of these events due to missing data or insufficient measurement quality. Other unclassified POFEs may have resulted from additional events not addressed here, or from cases in which the POFE1 and/or POFE2 played a role but the defining criteria (i.e., RH < 30% or FNO < 0) could not be identified using the 30 min averaging interval applied in our analysis.

Table S2 compares FO_3_, O_3_ mixing ratios, Vdw,O_3_, FNO, and FNO_2_ individually for POFE1s, POFE2s, all observed POFEs and all data. The table indicates that both POFE1s and POFE2s were associated with positive daytime FO_3_ values of a similar order of magnitude, with calculated daytime mean values of 4.83 and 5.24 nmol m^–2^ s^–1^, respectively. Only a single POFE1 was detected during the night, which showed a lower positive FO_3_ value than the mean nighttime FO_3_ for POFE2s. Mean daytime Vdw,O_3_ was −0.29 cm s^–1^ for POFE2s and −0.16 cm s^–1^ for POFE1s. In addition, Table S2 shows that the mean negative FNO during POFE2s had a larger absolute magnitude than the corresponding mean of all measured data during the daytime (−2.17 vs 1.77 nmol m^–2^ s^–1^) and nighttime (−0.88 vs 0.32 nmol m^–2^ s^–1^). In contrast, the mean measured FNO_2_ during POFE2s was also negative during both day and night but was smaller in absolute value compared to the corresponding values calculated for all observations.

### 3.2 The Effect of HAMNC Associated with NO Emissions from Elevated Sources

Increased NO concentrations above the EC measurement height generally reduce the measured Vdw,O_3_ through enhanced NO–O_3_ titration. A particular case occurs when these concentrations exceed those below the EC measurement height (HAMNC), which can produce reversals in the measured NO and O_3_ flux directions (POFE2). As discussed in Section 3.1, the observed NO–NO_2_–O_3_ relationships and the supporting analyses presented in Section S2.2 indicate that HAMNC formation is likely associated with NO emissions from elevated combustion sources located approximately 5–21 km from the measurement site (with one additional source at 33 km), under favorable transport conditions, while concurrent near-ground NO emissions can partially mask their contribution to the measured net FNO. To further examine this influence, we analyzed the relationships between FNO and Vdw,O_3_ considering the contributions of both elevated and near-ground NO emissions.

Table 2 presents the measured FO_3_, Vdw,O_3_, FNO, and FNO_2_, both including and excluding POFEs associated with negative FNO (POFE2) or dry conditions (RH < 30%; POFE1). Excluding data associated with POFE2s resulted in an average daytime FO_3_ that was 2.1% more negative (−11.17 vs −10.94 nmol m^–2^ s^–1^), and a 2.6% increase in Vdw,O_3_ (0.40 vs 0.39 cm s^–1^). Mean FNO and FNO_2_ also increased slightly (by 2.8% and 1.4%, respectively), because these POFE2s were associated with negative FNO and, in most cases, negative FNO_2_ (see Table 1). The negative FNO_2_ arises because HAMNC is generally accompanied by higher NO_2_ concentrations above the EC measurement height than below it, resulting in a downward NO_2_ flux.

**Table 2:** Mean and Standard Error of the Mean (SEM) for the Fluxes of O_3_ (FO_3_), NO (FNO), and NO_2_ (FNO_2_) and for Downward O_3_ Velocity (Vdw,O_3_), Presented Separately for Daytime and Nighttime, for the Complete Dataset and for the Dataset Excluding Events Classified as POFE1 or Those Classified as POFE2

Data	D/N	Measures	FO_3_ (nmol m^–2^ s^–1^)	Vdw,O_3_ (cm s^–1^)	FNO (nmol m^–2^ s^–1^)	FNO_2_ (nmol m^–2^ s^–1^)
All data	Day	Mean	–10.94	0.39	1.77	12.49
		SEM	0.32	0.01	0.08	0.23
	Night	Mean	–5.07	0.33	0.32	6.79
		SEM	0.43	0.10	0.13	0.42
Without POFE2	Day	Mean	–11.17	0.40	1.82	12.66
		SEM	0.31	0.01	0.07	0.23
	Night	Mean	–5.67	0.36	0.40	7.32
		SEM	0.42	0.11	0.13	0.40
Without POFE1	Day	Mean	–11.60	0.41	1.78	12.52
		SEM	0.31	0.01	0.08	0.24
	Night	Mean	–5.11	0.33	0.32	6.79
		SEM	0.43	0.11	0.13	0.41

Figure 1 shows the mean FO_3_, Vdw,O_3_ and the proportion of POFEs across FNO ranges, with and without POFE1 data, separately for day and night, with the corresponding numerical data summarized in Table S3. FO_3_ did not exhibit a consistent response to FNO, likely due to its dependence on O_3_ mixing ratios. In contrast, Vdw,O_3_, normalized by O_3_ concentration, displayed a consistent positive correlation with FNO during both daytime (*p* ≪ 0.005) and nighttime (*p* ≈ 0.001).

**Figure 1:**
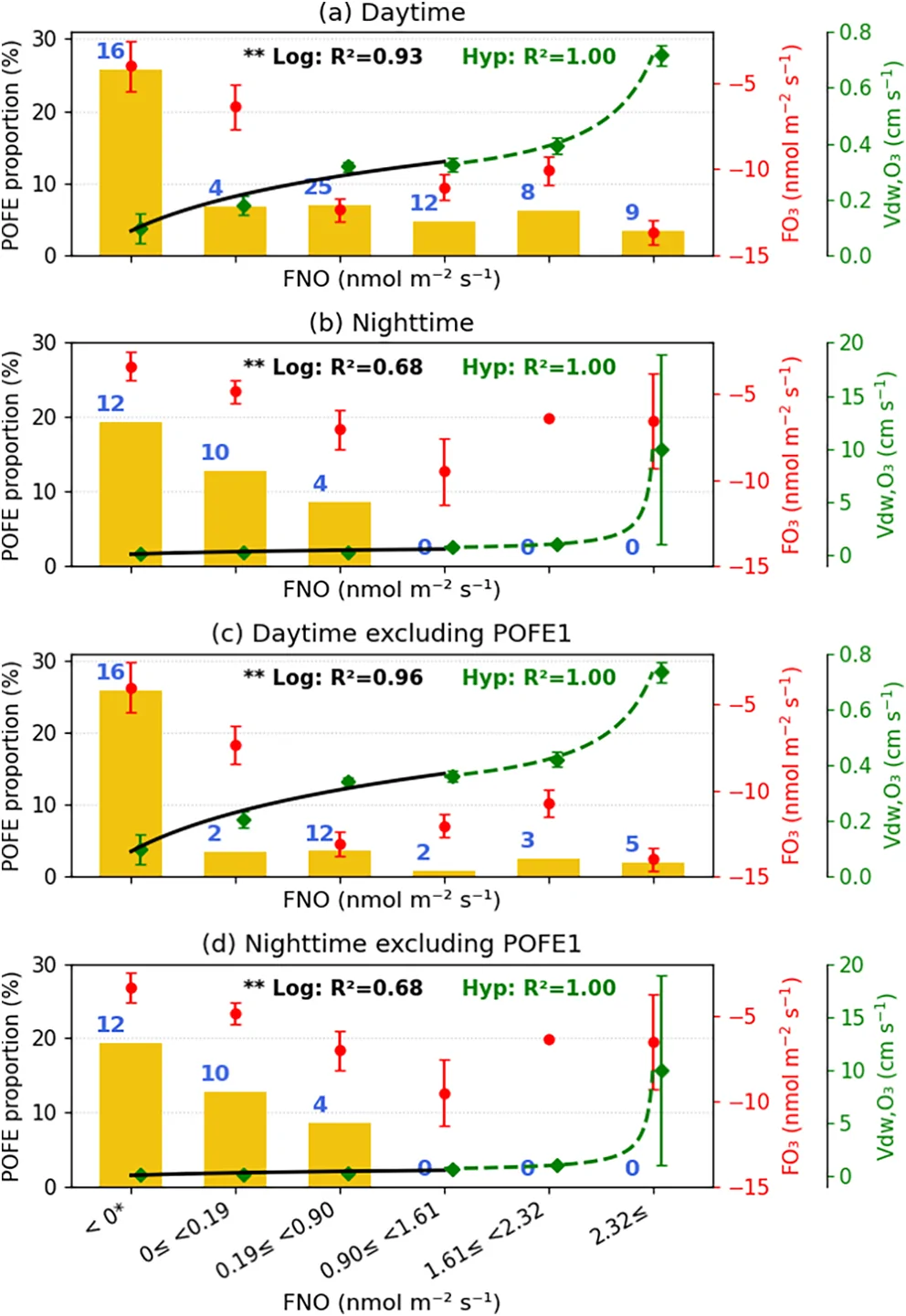
Ozone flux (FO_3_; red) and downward O_3_ velocity (Vdw,O_3_; green) along with the proportion of positive ozone-flux events (POFEs; gold bars) across NO flux (FNO) ranges during (a) daytime, (b) nighttime, (c) daytime without POFE1s and (d) nighttime without POFE1s. The number of POFEs is indicated above each bar in blue. The Vdw,O_3_–FNO relationships were fitted using two different functions: logarithmic (Log) and hyperbolic (Hyp), separated at FNO = 1.61 nmol m^–2^ s^–1^. Filled circles and diamonds represent mean values, and vertical bars indicate SEM. *The average FNO in the first bin (FNO < 0) is (a) −0.97, (b) −0.31, (c) −0.98, (d) −0.31 nmol m^–2^ s^–1^. **The mathematical definitions of the Log and Hyp functions, along with detailed fitting results, are provided in the SI (see Figure S4).

The observed positive correlation between FNO and Vdw,O_3_ reflects opposing contributions from different NO sources: near-ground NO emissions tend to increase Vdw,O_3_, whereas NO emissions from elevated sources can lead to HAMNC, which tends to reduce Vdw,O_3_ (Section S2.2). The measured FNO represents the net flux (i.e., the sum of contributions from elevated and near-ground sources), which is simultaneously affected by these opposing effects.

Therefore, these contributions cannot be separated into distinct upward (near-ground) and downward (associated with elevated sources) components using our measurements. Nevertheless, for the purpose of this analysis, we assume that the measured net FNO and its correlation with Vdw,O_3_ arise from a single dominant source—either near-ground NO emissions or NO emissions from elevated sources, which under suitable conditions can lead to HAMNC and negative FNO values. Accordingly, the logarithmic trend in the response ratio $\frac{\Delta \left(\mathrm{Vdw},O_{3}\right)}{\Delta \left(\mathrm{FNO}\right)}$ for increasing FNO up to approximately 1.61 nmol m^–2^ s^–1^ during the day can be attributed to emissions from near-ground sources. A logarithmic relationship is also maintained within the negative FNO range (∼−1.94 nmol m^–2^ s^–1^ to 0 nmol m^–2^ s^–1^; Figure S3), which probably reflects the decreasing influence of HAMNC as FNO increases toward zero.

Considering that near-ground NO emissions can simultaneously increase Vdw,O_3_, this logarithmic relationship can be regarded as a lower-limit estimate of the effect of HAMNC, likely associated with NO emissions from elevated sources, on Vdw,O_3_ in the urban environment of Beijing. At night, the logarithmic trend is less consistent than during the day (*R* ^2^ = 0.68 vs 0.93, or 0.96 after excluding POFE1s), likely reflecting the more stable nighttime atmosphere, which may disrupt the transport of NO between the emission source and the measurement point.

For FNO between ∼1.61 and 2.32 nmol m^–2^ s^–1^, $\frac{\Delta \left(\mathrm{Vdw},O_{3}\right)}{\Delta \left(\mathrm{FNO}\right)}$ exhibits a hyperbolic increase during both day and night (Figures 1, S4). Figure 1 shows that the slope $\frac{\Delta \left(\mathrm{Vdw},O_{3}\right)}{\Delta \left(\mathrm{FNO}\right)}$ is substantially larger at night (Figure 1b) than during the day (Figure 1a) in the hyperbolic regime (1.61–2.32 nmol m^–2^ s^–1^), suggesting that near-ground NO emissions enhance Vdw,O_3_ more effectively at night under stable conditions with a shallower mixing height than during the day.

Excluding data associated with the POFE1s (Figure 1c,d) did not affect the nighttime $\frac{\Delta \left(\mathrm{Vdw},O_{3}\right)}{\Delta \left(\mathrm{FNO}\right)}$ , and only slightly affected values associated with FNO < 1.61 nmol m^–2^ s^–1^ during the day, resulting in an improved logarithmic fit (*R* ^2^ = 0.96 vs 0.93). The increase in *R* ^2^ suggests that POFE1s tend to mask the influence of FNO on Vdw,O_3_ during the daytime, when they were more frequent, under generally lower RH.

Figure 1 shows that during the daytime, the proportion of POFEs was significantly higher for the negative FNO range than for the non-negative FNO ranges (*p* ≪ 0.005), with no clear trend for positive FNO, regardless of whether POFE1s were excluded from the analysis (Figure 1c,d) or included in it (Figure 1a,b). Notably, several daytime POFEs were also associated with high positive FNO ranges even after excluding POFE1-related data. This may reflect cases in which RH < 30%, negative FNO, or both, occurred for periods shorter than 30 min within the averaging interval, and thus were not captured by the 30 min mean values shown in Figure 1. Such subinterval events could have influenced FO_3_ direction even when the 30 min mean FNO remained positive and RH exceeded 30%.

Unlike during the day, the proportion of POFEs decreased more consistently with increasing FNO at night. This more consistent nighttime response may reflect the greater stability of the nighttime boundary layer, which reduces vertical mixing and limits the dispersion of elevated plumes during transport to the measurement point, while near-surface NO emissions are reduced according to the local traffic index (Figure S7),^18^ thereby producing a larger vertical NO concentration difference across the EC measurement height.

Overall, HAMNC, likely associated with NO emissions from elevated sources, can substantially affect Vdw,O_3_ measured at the EC height through NO–O_3_ chemistry. Given the widespread occurrence of elevated emission sources in urban environments, elevated-source NO emissions may affect measured O_3_ fluxes even under conditions that do not result in HAMNC. NO–O_3_ chemistry associated with elevated NO emissions may also contribute to chemical O_3_-flux divergence between the surface and the EC measurement height, such that the O_3_ flux measured at 103 m may differ from the actual surface O_3_ flux. However, because O_3_ flux was measured only at 103 m, such chemical flux divergence cannot be quantified from our observations. An order-of-magnitude analysis (see Section S5) further suggests that horizontal advection may be sufficiently large to contribute to the vertically integrated O_3_ budget and therefore has the potential to contribute both to the occurrence of positive O_3_ fluxes at the EC measurement height and to a difference between the surface and 103-m fluxes, although the magnitude and direction of its actual effect cannot be determined from our observations.

More broadly, interpretation of urban O_3_ flux measurements should not be limited to near-ground NO emissions, but should also explicitly consider NO emissions from elevated sources. Importantly, the influence of such elevated emissions may not be readily identified from routine ground-based measurements alone and can therefore complicate the attribution of processes controlling measured urban O_3_ fluxes. For model–measurement comparisons, models should likewise adequately represent both near-ground and elevated NO emissions, together with horizontal advection and vertical mixing, which influence the spatial distribution of NO and O_3_ and thereby the extent to which NO–O_3_ chemistry affects urban O_3_ fluxes.

### 3.3 Effect of Air Dryness on O_3_ Dry Deposition

#### 3.3.1 Effect of RH on Vdw,O_3_ and POFE Occurrence

We found that decreasing RH strongly suppresses Vdw,O_3_ and increases the frequency of POFEs under dry conditions. Current understanding of O_3_ dry deposition to urban surfaces is limited, and most knowledge of this process is derived from studies of O_3_ deposition to vegetation.^12^ Nevertheless, a chamber study of common urban materials, complemented by a review of the available literature, showed that O_3_ uptake generally increases with relative humidity for most tested urban surface materials across the investigated RH range (0–90%), including metals, glass, and surface-treated building materials.^36^ In contrast, porous structural materials such as concrete and limestone exhibited reduced deposition efficiency with increasing RH, which was attributed to differences in chemical composition, gas permeability, and substrate porosity.

Understanding of ozone uptake by urban surfaces can be informed by extensive knowledge gained from studies of O_3_ deposition to vegetation.^12^ Although O_3_ is relatively insoluble, its uptake by wet vegetated surfaces can become significantly more efficient following the formation of a waxy layer, along with salts and other oxidation products on the leaf surface.^37–45^ Changes in RH affect leaf wetness, thereby affecting the O_3_ uptake rate by leaves. For example, a hyperbolic decrease in nonstomatal resistance has been reported at RH values above approximately 70–80% ^37,45^ whereas under very dry conditions (RH < 25%), Li et al.,^19^ based on flux measurements in the semiarid Yatir Forest, repeatedly observed a reversal in FO_3_ direction. These very dry conditions are generally associated with both low atmospheric humidity and dry surface or soil,^46^ indicating strongly suppressed surface O_3_ uptake.^19^

Based on measurements at the IAP, Choi et al.^18^ reported that the response of Vdw,O_3_ to RH follows three distinct regimes: (i) a logarithmic increase for RH_8m_ < 30% (RH-low), (ii) a minor and inconsistent increase for 30% ≤ RH_8m_ < 70% (RH-mid) and (iii) a hyperbolic increase for RH_8m_ ≥ 70–80% (RH-high). Figure 2 presents Vdw,O_3_ as a function of binned RH_8m_ for the entire data set (Figure 2a) and separately for RH_8m_ < 30% (Figure 2b) and RH_8m_ ≥ 70% (Figure 2c) during the daytime.^18^ Two clear regimes are observed: a logarithmic increase for RH-low (RH_8m_ between 5% and 30%; Figure 2b, *R* ^2^ = 1.0) and a hyperbolic increase for RH-high (RH_8m_ between 70% and 97%; Figure 2c, *R* ^2^ = 0.97). The mean response of Vdw,O_3_ to RH_8m_, (ΔVdw,O_3_)/(ΔRH_8m_), is $0.02\;\frac{\mathrm{cm}}{s\cdot \%}$ for RH-low and $0.07\;\frac{\mathrm{cm}}{s\cdot \%}$ for RH-high, while the RH-mid range shows a much weaker and less consistent response, with a mean of $0.002\;\frac{\mathrm{cm}}{s\cdot \%}$.

**Figure 2:**
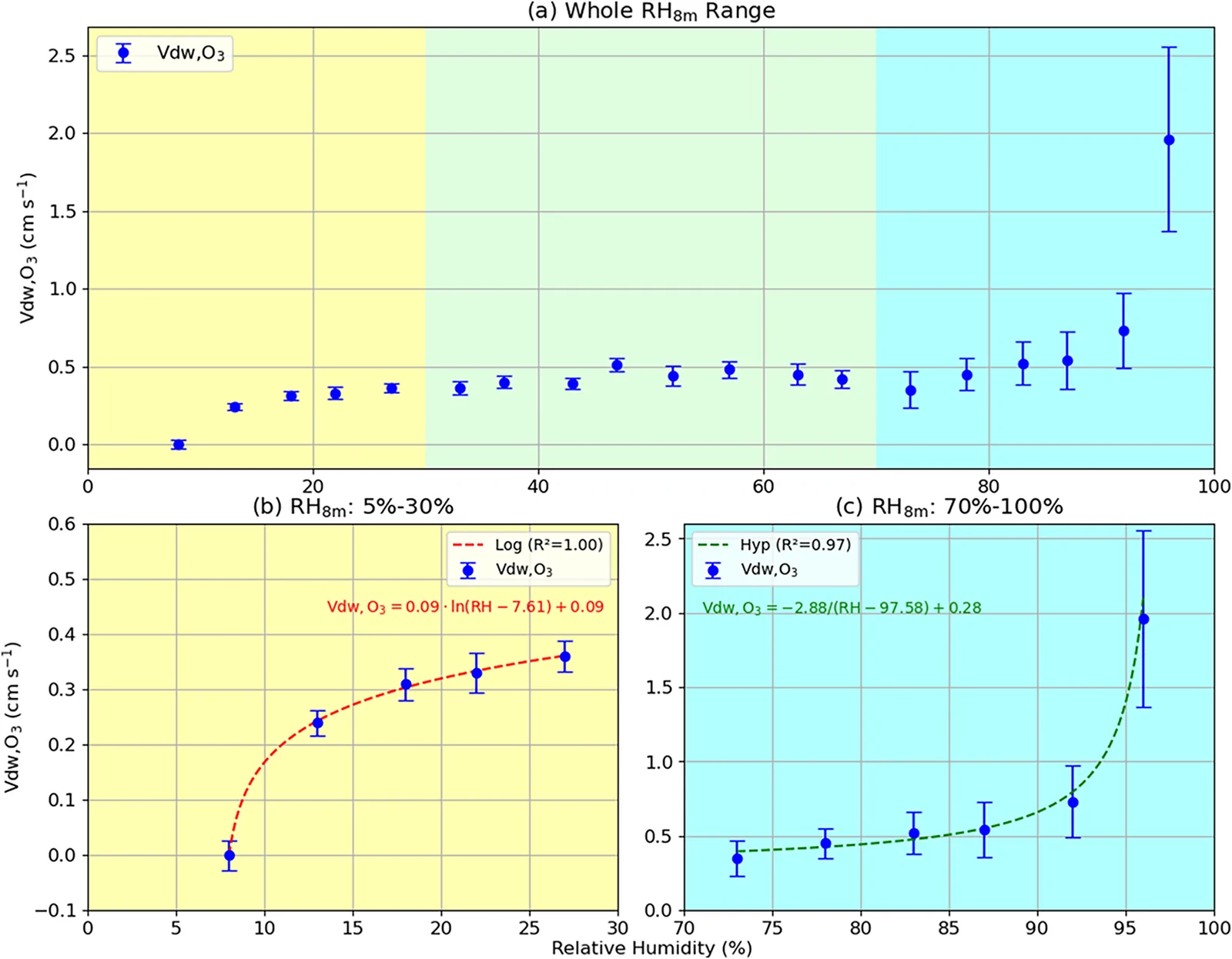
Response of downward ozone velocity (Vdw,O_3_) to changes in relative humidity (RH) during the daytime. (a) Full RH_8m_ range with mean Vdw,O_3_ shown as blue circles and vertical bars indicating SEM; background shading highlights three RH_8m_ regimes: low (<30%, yellow), intermediate (RH-mid; 30–70%, light green) and high (≥70%, cyan). (b) low RH_8m_ regime (0–30%) with a logarithmic fit (Log; dashed red line). (c) high RH_8m_ regime (70–100%) with a hyperbolic fit (Hyp; dashed green line). Fits and R^2^ values shown in each panel correspond to the displayed curves.

Figure 3 presents the proportion of POFEs, FO_3_ and Vdw,O_3_ across different RH_8m_ ranges, showing all measurements (Figure 3a) and the data after excluding POFE2s (Figure 3b). The figure indicates that FO_3_ becomes consistently less negative with decreasing RH_8m_ within the RH-low regime, demonstrating an overall reduced uptake of O_3_ by the surface under low RH_8m_ (Table S4a). This resulted from decreases in both O_3_ mixing ratio (see Figure S10) and Vdw,O_3_ as RH_8m_ decreased within RH-low. The figure further shows a higher mean proportion of POFEs for RH-low compared to RH-mid and RH-high, more notably after excluding the POFE2s (Figure 3b). The mean proportion of POFEs under RH-low (RH < 30%) was 10.11%, compared to 7.26% for RH_8m_ ≥ 30% (Figure 3a); this difference was not statistically significant (*p* = 0.078; see Table S4a). After excluding POFE2-associated observations, the difference became more pronounced: the proportion of POFEs for RH_8m_ < 30% was 9.91%, compared to 5.06% for RH_8m_ ≥ 30%; this difference was statistically significant (*p* = 0.002; see Table S4b).

**Figure 3:**
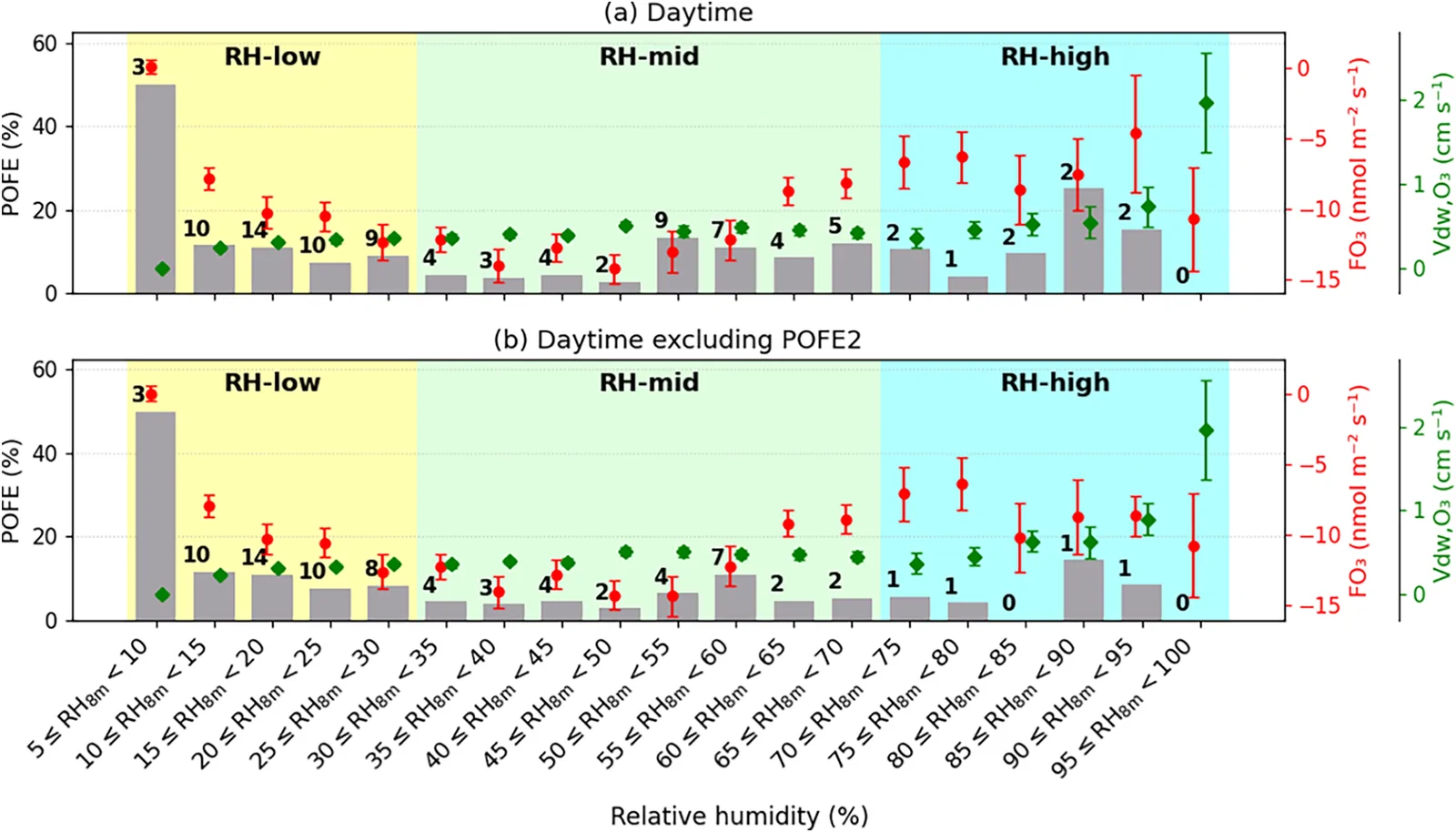
Ozone flux (FO_3_; red) and downward O_3_ velocity (Vdw,O_3_; green) plotted with the proportion of positive ozone-flux events (POFEs; gray bars) across relative humidity (RH) bins for (a) daytime and (b) daytime excluding observations associated with POFE2s. Background shading denotes RH8m ranges: RH-low (5–30%, yellow), RH-mid (30–70%, light green) and RH-high (70–100%, cyan). Numbers above the gray bars indicate the count of POFEs in each bin. Filled circles and diamonds represent mean values, and vertical bars indicate SEM.

While Vdw,O_3_ exhibited a consistent logarithmic increase with RH_8m_ within the RH-low range, the proportion of POFEs in this regime did not show a clear, statistically significant trend with RH_8m_, although there was a weak tendency of POFE frequency to decrease as RH_8m_ increased. The increase of Vdw,O_3_ with RH_8m_ within RH-low reflects a strong decrease in surface resistance as RH_8m_ rises. However, a POFE depends on the difference between surface resistance at the ground and that aloft (near the measurement point), and therefore does not directly correspond to the near-ground RH_8m_ or show a clear association with it.

The apparently lower surface resistance aloft compared to the ground, associated with POFE1s, may reflect the relatively higher RH aloft and/or larger aerosol surface area and altered surface properties aloft, which may enable more efficient uptake and thus a stronger O_3_ sink aloft than near the ground. However, our analysis did not reveal a distinctly less negative vertical RH gradient between 8 and 103 m above the ground during POFE1s (see Figure S2). Aerosols, particularly those of anthropogenic origin, may have relatively low deliquescence points due to uptake of hygroscopic salts such as MgCl_2_,^47–50^ which could enhance heterogeneous uptake and thus facilitate a more efficient O_3_ sink aloft than near the ground under dry conditions. Additional processes, including gas-phase reactions of O_3_ with NO and VOCs above the EC measurement height, may further contribute to O_3_ removal aloft, thereby increasing the likelihood of POFE1 occurrence.

#### 3.3.2 Role of Surface Evaporation in Controlling Vdw,O_3_

The effect of RH on Vdw,O_3_ via its influence on surface resistance may also involve other meteorological parameters that promote surface evaporation, and thus surface drying, which in turn increases resistance to O_3_ deposition. To analyze meteorological controls on POFE1, we examined the dependence of Vdw,O_3_ on meteorological parameters other than RH that govern surface wetness through evapotranspiration and thereby influence Vdw,O_3_ and the frequency of POFEs. According to the Penman–Monteith equation,^51^ evapotranspiration is controlled by WS, solar radiation, RH and T. Accordingly, Figure 4 presents a principal component analysis (PCA) that investigates the correlations between Vdw,O_3_ and these meteorological parameters, together with VPD and friction velocity (u_*_), for individual RH ranges, whereas Figure S11 shows the same analysis after excluding data associated with POFE2s. The PCA was conducted separately for different RH ranges to examine how the relationships between Vdw,O_3_ and the meteorological variables vary across RH conditions representing different levels of surface dryness.

**Figure 4:**
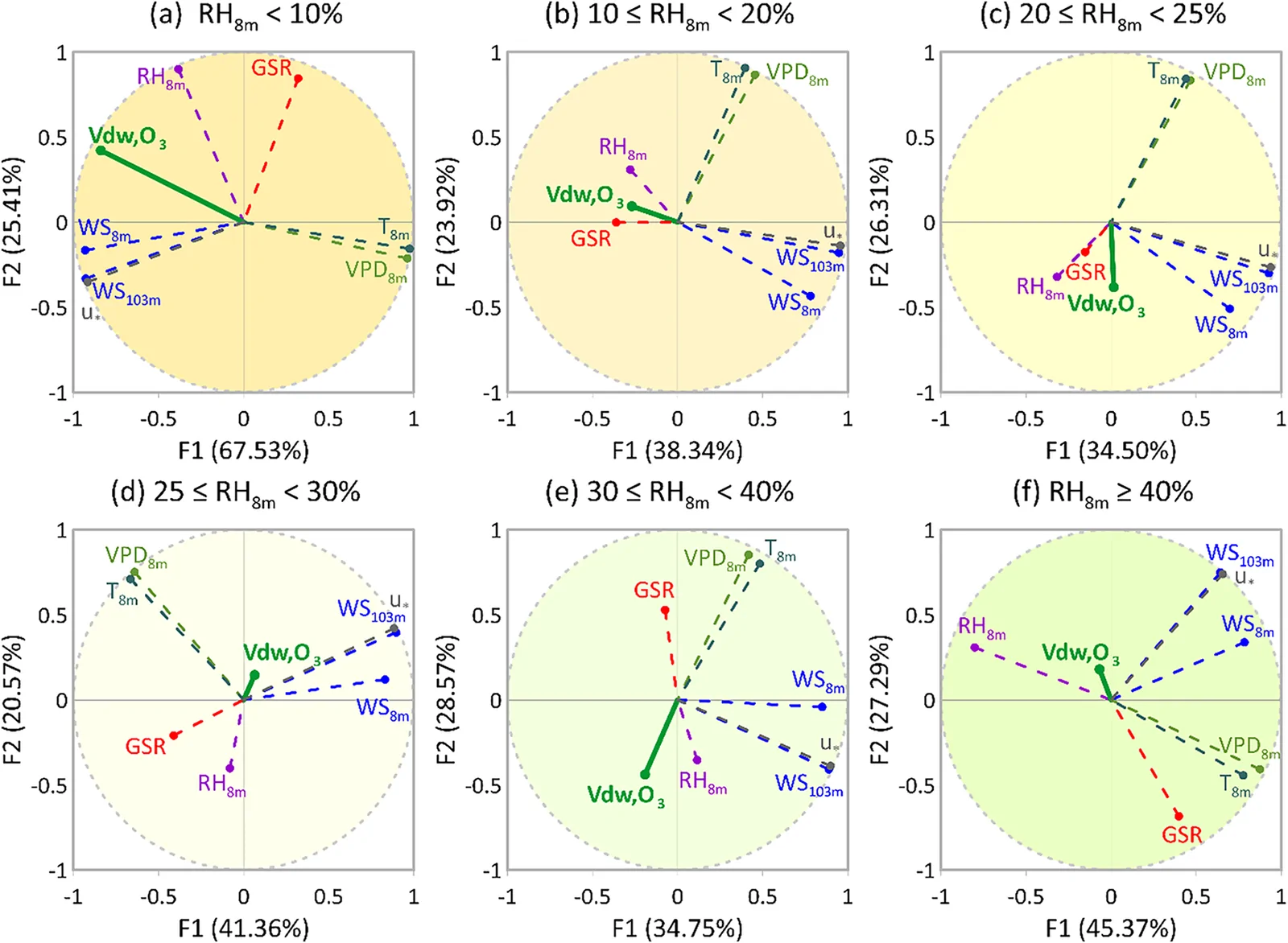
PCA results for daytime conditions (defined as periods with positive global solar radiation) based on half-hourly observations across relative humidity (RH) ranges: (a) RH_8m_ < 10%, (b) 10% ≤ RH_8m_ < 20%, (c) 20% ≤ RH_8m_ < 25%, (d) 25% ≤ RH_8m_ < 30%, (e) 30% ≤ RH_8m_ < 40% and (f) RH_8m_ ≥ 40%. Abbreviations: Vdw,O_3_ (downward velocity of ozone), GSR (global solar radiation), T (temperature), VPD (vapor-pressure deficit), WS (wind speed) and u_*_ (friction velocity). See Section 2.1 for details on meteorological parameter elevation indexing. F1 and F2 denote the first and second principal components, respectively. Percentages in parentheses indicate the proportion of total variance explained by each principal component.

Overall, Figure 4 supports an influence of RH_8m_, VPD_8m_, and *T*_8m_ on Vdw,O_3_. Vdw,O_3_ covaried positively with RH_8m_ and negatively with *T*_8m_ and VPD_8m_ for 30% ≤ RH_8m_ < 40%, but not for 25% ≤ RH_8m_ < 30% (for RH_8m_) or for 10% ≤ RH_8m_ < 20% and 25% ≤ RH_8m_ < 30% (for *T*_8m_ and VPD_8m_). These inconsistencies may reflect the effect of global solar radiation (GSR) on Vdw,O_3_. Notably, GSR and Vdw,O_3_ covaried positively most strongly in the 10%–20% RH_8m_ range—a range in which RH_8m_, VPD_8m_ and *T*_8m_ exhibited relatively weak associations with Vdw,O_3_ compared to the other ranges (Figures 4 and S13, Table S5). The positive GSR–Vdw,O_3_ correlation consistently weakened with increasing RH_8m_, from RH_8m_ ≥ 10% to RH_8m_ ≥ 40%, turning negative for RH_8m_ ≥ 25%.

The systematic shift of the GSR–Vdw,O_3_ relationship—from a strong positive covariance at 10%–25% RH_8m_ to a strong negative covariance for RH_8m_ ≥ 30% (Figure 4)—suggests that GSR influences Vdw,O_3_ through two distinct mechanisms. One possible explanation is that strong GSR enhances near-surface turbulence and mixing, which could potentially increase Vdw,O_3_ under very low RH conditions that are typically associated with high radiation and temperature. However, under well-mixed daytime conditions, the effect of turbulence on Vdw,O_3_ is expected to be limited.^52^ Consistent with this expectation, the PCA (Figure 4) indicates that WS_8m_, WS_103m_ and u_*_ did not exhibit a strong positive covariance with Vdw,O_3_, particularly in the 10–20% RH_8m_ range, where their loadings indicate negative covariance with Vdw,O_3_. A distinctly positive covariance with Vdw,O_3_ was observed only under extremely dry conditions (RH_8m_ < 10%; Figure 4a and Figure S13).

An additional possible explanation for the strong GSR–Vdw,O_3_ covariance observed in the 10–20% and 20–25% RH_8m_ ranges is that GSR alters surface properties and/or temperature in ways that enhance surface reactivity, thereby increasing conductance to O_3_.^53,54^ Coyle et al.^53^ suggested that, under very dry conditions, nonstomatal deposition of O_3_ to a potato canopy may involve thermal decomposition of O_3_ at the leaf surface, with the O_3_ removal rate increasing with surface temperature and potentially being further enhanced during daytime by photolytic reactions driven by solar radiation. In contrast, at higher RH (e.g., 50–70% in previous studies), a thin liquid film suppresses this process.^53,54^ Given that O_3_ uptake at the surface was found to consistently increase with temperature for various surface types,^55^ a higher solar radiation flux could also enhance O_3_ uptake simply via temperature increases—particularly under dry conditions. While urban surfaces may behave differently from vegetated canopies, the general mechanisms of the nonstomatal pathway are likely to be similar. This mechanism is proposed as a plausible additional pathway but is not directly demonstrated by the present data set.

The transition to a negative GSR–Vdw,O_3_ covariance for RH_8m_ ≥ 30% can be directly related to GSR-driven surface evaporation. Higher radiation dries wet or hygroscopic surface films, reducing surface wetness and lowering surface conductance, which is dominated by nonstomatal pathways, and consequently decreases Vdw,O_3_. The transition may also arise through a more indirect pathway, whereby increased GSR raises near-surface O_3_ mixing ratios—by either enhancing photochemical production or increasing mixing height and vertical transport of O_3_ from aloft.^56–59^ For instance, Fallmann et al.^57^ using a multilayer urban-canopy model coupled to WRF-Chem, demonstrated that changes in shortwave reflectance by urban surfaces can increase surface O_3_; their study reported a corresponding increase of up to ∼12% under certain scenarios. When fluxes are limited by surface resistance, an increase in near-surface O_3_ concentration without a proportional increase in FO_3_ can be reflected in a lower Vdw,O_3_.

Figure 4 does not conclusively support the hypothesis that surface evaporation decreases Vdw,O_3_ by drying the surface, because it does not show consistent covariation between Vdw,O_3_ and the principal drivers of evaporation: GSR, RH_8m_, *T*_8m_, and WS. This may be because the effect of surface evaporation is masked by additional effects of GSR on Vdw,O_3_, as discussed above. Similarly, it is not possible to determine whether WS reduced Vdw,O_3_ via enhanced surface evaporation, because that effect may have been obscured by the direct influence of wind-driven turbulence on Vdw,O_3_, as reported previously.^60–62^ Such evaporative effects could, however, be relatively more important under extremely dry conditions (RH_8m_ < 10%) (Figure 4a).

Because the correlations of the meteorological parameters with Vdw,O_3_ identified by the PCA in Figure 4 may be confounded by complex direct and indirect interactions, we conducted a more direct test of the relationships between Vdw,O_3_ and meteorological parameters that control surface evaporation. Figure 5 shows deviations from mean values of WS, u_*_, *T*_8m_, VPD_8m_, GSR and Vdw,O_3_ across RH_8m_ bins. To remain consistent with the definition of POFE1 and with the analysis in Figure 4, the thresholds of RH_8m_ ≥ 30% and RH_8m_ ≥ 40% are marked with red and yellow dashed lines, respectively. Data associated with RH_8m_ < 10%, for which wind-driven turbulence seems to play a more important role in affecting Vdw,O_3_ compared to other RH_8m_ ranges, are marked with a dashed black line.

**Figure 5:**
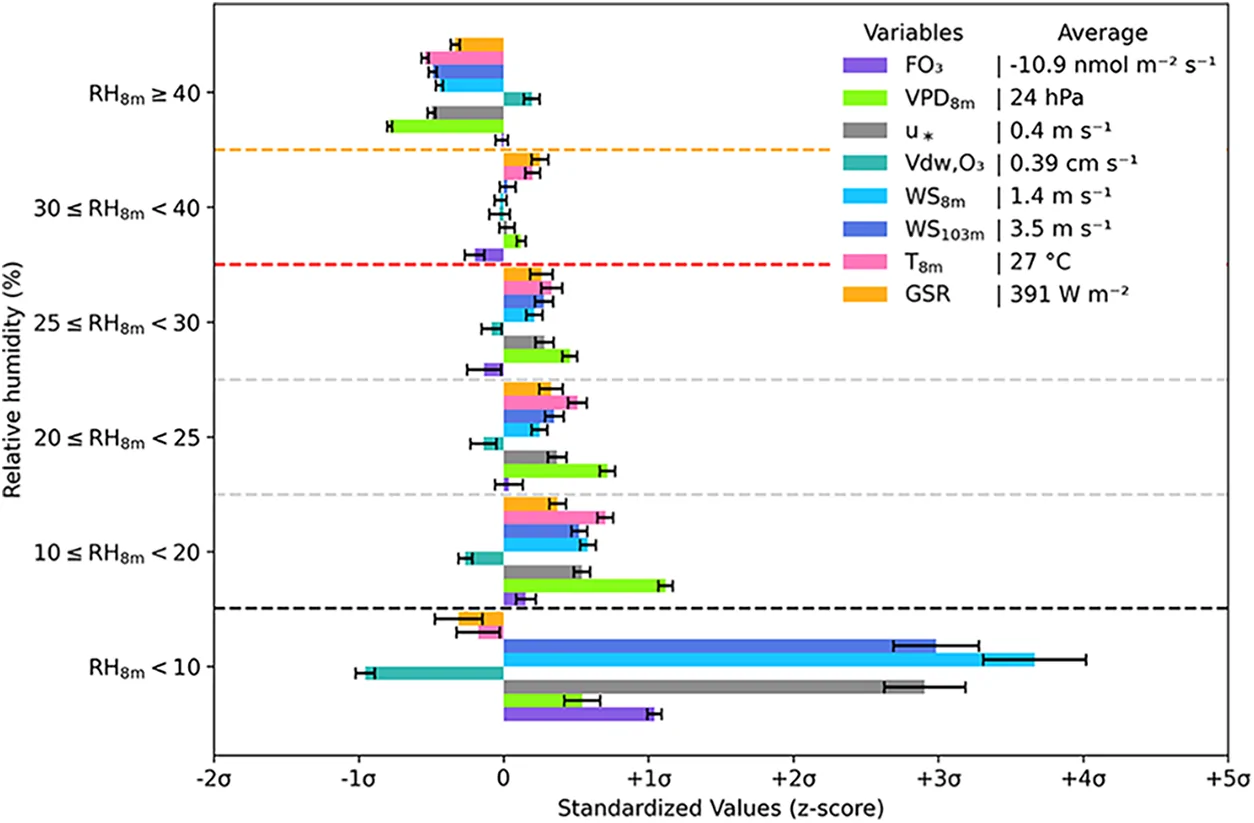
Standardized (*z*-score) profiles of O_3_ flux (FO_3_), downward velocity of ozone (Vdw,O_3_), wind speed (WS), temperature (*T*), vapor-pressure deficit (VPD), global solar radiation (GSR) and the friction velocity (u_*_) across the bin-based relative humidity (RH_8m_) ranges during the daytime. See Section 2.1 for details on the elevation indexing of meteorological parameters. Horizontal bars indicate the mean *z*-score for each RH bin (see Figure S14 for calculation); black ticks indicate SEM. Dashed red line shows the RH_8m_ threshold (<30%) used to define POFE1, and dashed orange line indicates an extra split within the RH_8m_ ≥ 30% range. Dashed black line shows RH_8m_ < 10%, where wind-driven turbulence apparently played a more important role in affecting Vdw,O_3_. See Figure S14 for an alternative visualization.

Figure 5 indicates that both WS_8m_ and u_*_ decline systematically across increasing RH_8m_ bins, while Vdw,O_3_ increases. This suggests that WS influences Vdw,O_3_ primarily via surface evaporation rather than through its direct effect on turbulence. VPD_8m_ did not show a consistent trend with Vdw,O_3_, possibly because VPD_8m_ is a function of both RH_8m_ and *T*_8m_, which tend to be anticorrelated. Notably, although *T*_8m_ and GSR tended to decrease with increasing RH bins, they were exceptionally lower in the RH_8m_ < 10% bin, whereas WS_8m_, WS_103m_ and u_*_ were exceptionally high in that bin. This suggests that WS played an important role in reducing Vdw,O_3_ for RH_8m_ < 10% by enhancing surface evaporation and therefore reducing surface wetness. This interpretation is consistent with Figure 4, which also indicates a relatively minor role of GSR and a larger role of WS in affecting Vdw,O_3_ at RH_8m_ < 10%.

Overall, the negative associations of *T*_8m,_ WS_8m_, and GSR with Vdw,O_3_, together with the positive association of RH_8m_ with Vdw,O_3_ for RH_8m_ ≥ 10% inferred from Figure 5, are consistent with the hypothesis that enhanced surface evaporation inhibits surface conductance by reducing surface wetness. This inhibition, combined with the higher frequency of POFE1s at low RH_8m_, suggests that POFE1s arise when the near-ground resistance to O_3_ uptake exceeds the resistance aloft, typically under RH_8m_ < 30%. Consistent with this interpretation, Li et al.^19^ showed that POFEs can be induced under low RH conditions (typically below 25%), in several cases associated with dry-air intrusions from the upper troposphere, which are characterized by low absolute humidity.

In our study, although vegetation accounted for only ∼16% of the investigated flux footprint, the response of nonstomatal Vdw,O_3_ to changes in RH_8m_ across RH-low, RH-mid and RH-high showed similar patterns to those reported for vegetated surfaces. This similarity suggests that comparable controls, with a consistent sensitivity to meteorological conditions, regulate nonstomatal Vdw,O_3_ over both urban and vegetated surfaces. Accordingly, a common functional form with land-use-specific parameter values may be applicable for representing surface uptake of O_3_ in air-quality and climate models across different land-use types.

At present, the effect of air dryness on surface uptake of O_3_ is missing in climate and air-quality models, potentially biasing simulated O_3_ removal and our understanding of O_3_ deposition in urban environments. Given that ∼40% of the global land surface is arid or semiarid, omission of this dryness effect could lead to misinterpretation of observed changes in O_3_ dry deposition across extensive geographic regions under dry conditions. We therefore recommend incorporating near-surface dryness controls into future modeling schemes to more accurately capture the magnitude of surface O_3_ uptake under dry conditions.

## Supplementary Material



## Notes

Observed positive ozone-flux events (POFEs): characteristics, frequency and effects (Section S1); evidence supporting the role of elevated emission sources in POFE2 occurrence through HAMNC formation (Section S2); effect of air dryness on O_3_ dry deposition (Section S3); relevance of NO_*x*_ and volatile organic compounds (VOCs) emissions to Vdw,O_3_: PCA-based covariance (Section S4); horizontal O_3_ advection sensitivity analyses (Section S5); O_3_ random-flux uncertainty assessment and robustness analyses (Section S6) (PDF)

## Notes

Correspondence regarding data analysis and scientific interpretation should be addressed to Eran Tas (eran.tas@mail.huji.ac.il); correspondence regarding field measurements and the experimental setup should be addressed to Bin Yuan (byuan@jnu.edu.cn).
