# Supplementary material for: Limitation of O3 Dry Deposition by Elevated-Source NO Emissions and Surface Dryness in Urban Environments
Source: Environ Sci Technol. 2026 Sep 22;60(39):27955–66. doi: 10.1021/acs.est.6c05093 (PMC13648240; doi:10.1021/acs.est.6c05093)
Supplement: Supplementary file 1 [file es6c05093_si_001.pdf]

## *Supporting Information*

# Limitation of $O_3$ Dry Deposition by Elevated-Source NO Emissions and Surface Dryness in Urban Environments

*Daniel Choi*[a,‡], *Yibo Huangfu*[b,‡], *Erick Fredj*[c], *Qian Li*[a,d], *Bin Yuan*[b,*], *Xiaoxiao Zhang*[b], *Xianjun He*[b], *Huizhi Liu*[e], *Eran Tas*[a,*]

[a] The Robert H. Smith Faculty of Agriculture, Food and Environment, Department of Soil and Water Sciences, The Hebrew University of Jerusalem, Rehovot 7610001, Israel

[b] College of Environment and Climate, Institute for Environmental and Climate Research, Guangdong-Hongkong-Macau Joint Laboratory of Collaborative Innovation for Environmental Quality, Jinan University, Guangzhou 511443, China

[c] Department of Computer Science, Jerusalem College of Technology, Jerusalem 91160, Israel

[d] School of Ecology and Environment, Hainan University, Haikou, Hainan province 570228, China

[e] Department of Atmospheric Sciences, Yunnan University, Kunming 650500, China

‡ These authors contributed equally.

*Correspondence to:

Eran Tas, Institute of Environmental Sciences, The Robert H. Smith Faculty of Agriculture, Food and Environment, The Hebrew University of Jerusalem, P.O. Box 12, Rehovot 7610001, Israel, eran.tas@mail.huji.ac.il

Bin Yuan, College of Environment and Climate, Institute for Environmental and Climate Research, Guangdong-Hongkong-Macau Joint Laboratory of Collaborative Innovation for Environmental Quality, Jinan University, Guangzhou 511443, China, byuan@jnu.edu.cn

## S1. Observed positive ozone-flux events (POFEs): characteristics, frequency and effects

To identify the location of the elevated emission sources associated with the type 2 POFEs (POFE2s), wind data were analyzed (see Sect. 3.1). Figure S1 shows that negative NO-flux events occurred mainly under west-southwesterly (WSW) to northwesterly (NW) winds at 240 m, with a similar west-sector tendency at 103 m.

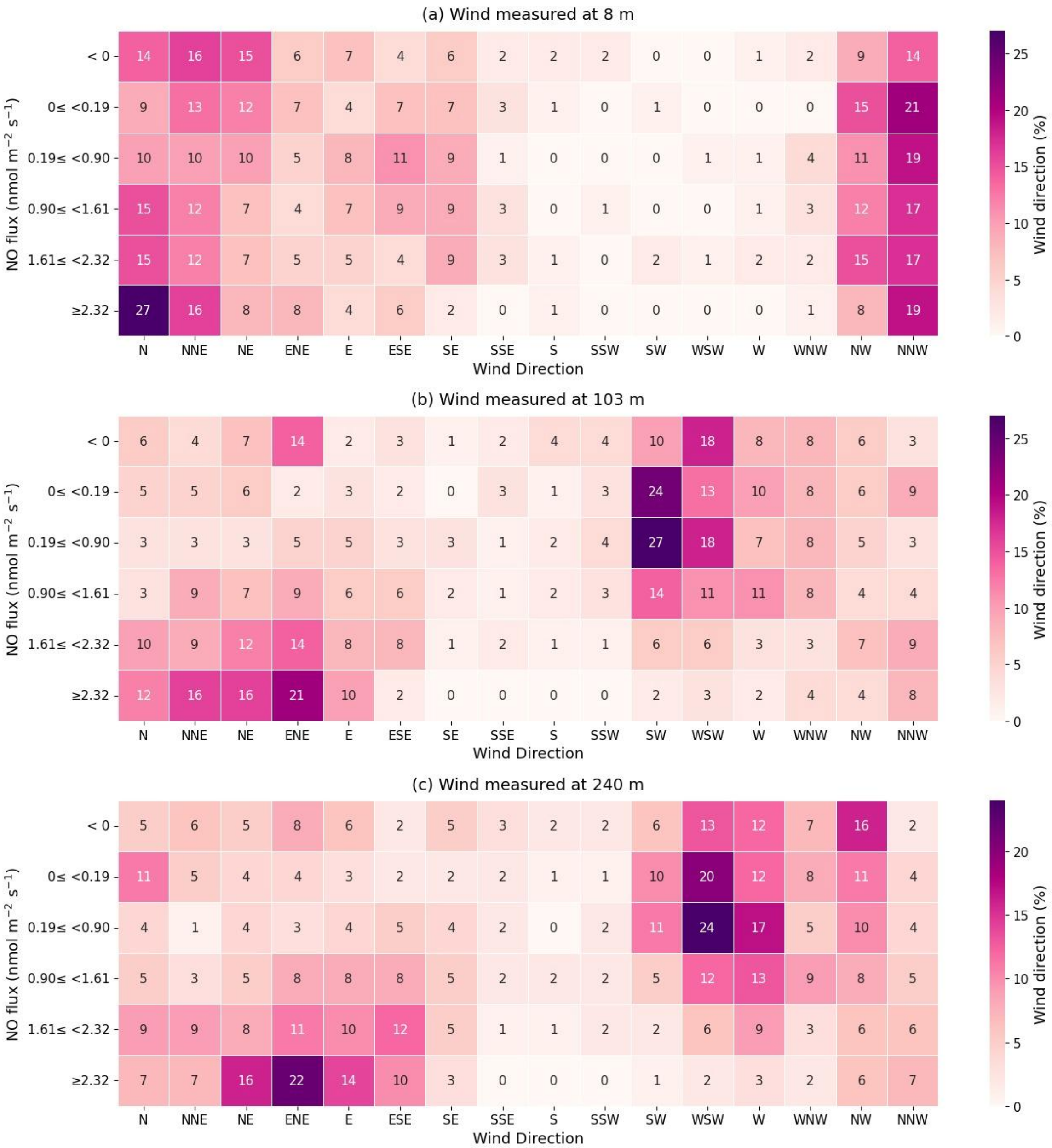


***Fig. S1.*** *Wind-direction frequency (%) for different NO-flux ranges at three measurement heights, using 16 wind-direction sectors. For each NO-flux bin, the numbers in the cells indicate the percentage of observations from each wind sector (rows sum to 100%), and the color shading is proportional to the corresponding frequency. Panels show wind directions measured at (a) 8 m (near ground), (b) 103 m (closest measurement height to the eddy-covariance system; EC height: 102 m), and (c) 240 m above ground level.*

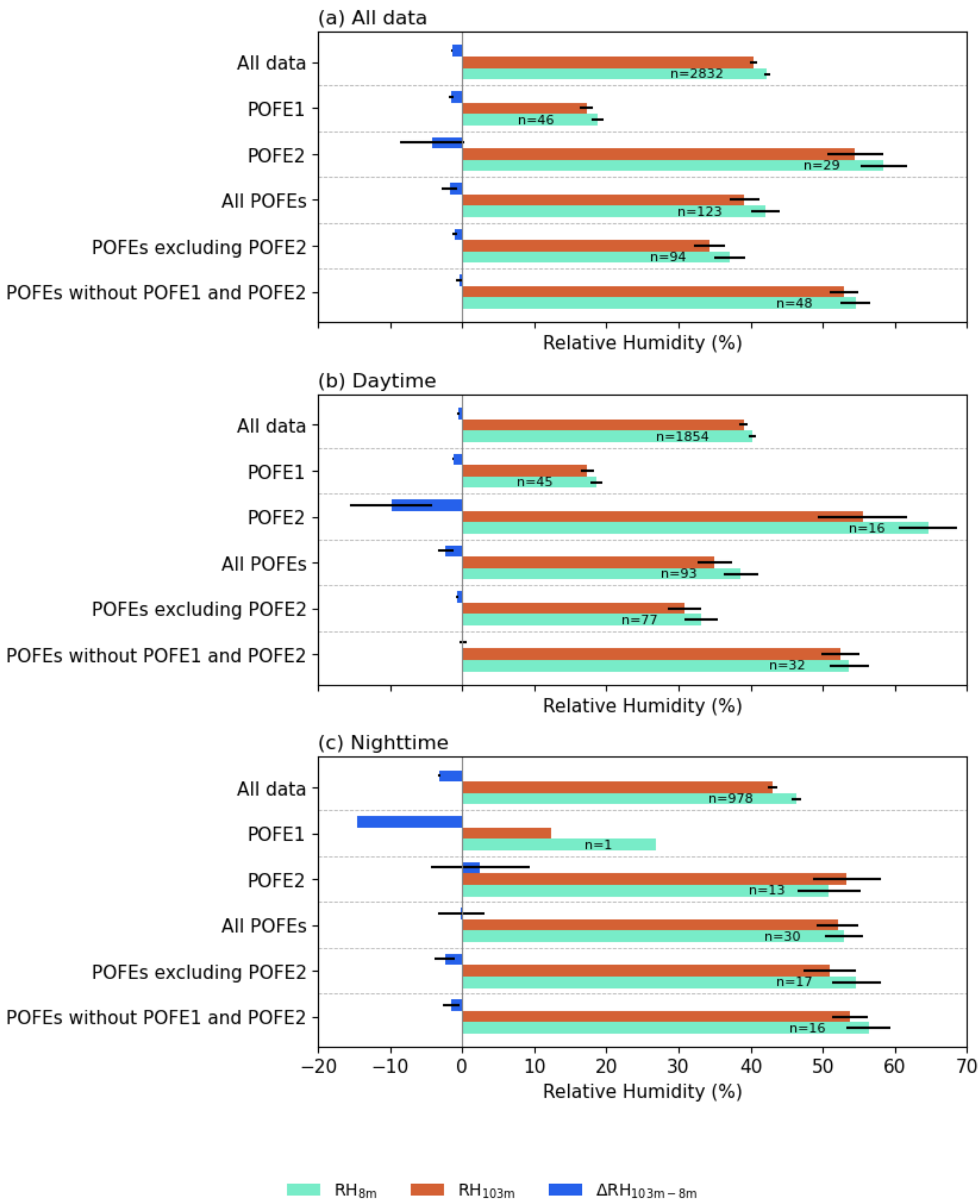


***Fig. S2.*** *Mean relative humidity at 8 m ($RH_{8m}$, green bars) and 103 m ($RH_{103m}$, orange bars), and the vertical difference in RH between the two levels ($\Delta RH_{103m-8m} = RH_{103m} - RH_{8m}$, blue bars) for different positive ozone-flux event (POFE) categories (all data, POFE1, POFE2, all POFEs, POFEs excluding POFE2, and POFEs without POFE1 and POFE2). Panels show (a) all data, (b) daytime, and (c) nighttime. Bars indicate the mean RH, black ticks indicate standard error of the mean (SEM). Numbers (n) on each bar show the number of available data records in each subset.*

***Table S1***

*Mean relative humidity (RH; see Sect. 2.1 of the main text) and its vertical difference (ΔRH = $RH_{103m}$ − $RH_{8m}$) for different positive ozone-flux event (POFE) categories and time periods (all data, day, night). Values in parentheses indicate the number of available data records in each subset. ΔRH was calculated as the mean of the paired differences.*

| **All data** | | | |
|---|---|---|---|
| **Category** | **$RH_{8m}$ (%)** | **$RH_{103m}$ (%)** | **ΔRH (%)** |
| **All data (2832)** | 42.3 | 40.4 | -1.5 |
| **POFE1 (46)** | 18.8 | 17.2 | -1.6 |
| **POFE2 (29)** | 58.5 | 54.5 | -4.1 |
| **All POFEs (123)** | 42.1 | 39.1 | -1.8 |
| **POFEs excluding POFE2 (94)** | 37.1 | 34.3 | -1.0 |
| **POFEs without POFE1 and POFE2 (48)** | 54.6 | 52.9 | -0.4 |
| **Day** | | | |
| **Category** | **$RH_{8m}$ (%)** | **$RH_{103m}$ (%)** | **ΔRH (%)** |
| **All data (1854)** | 40.2 | 39.1 | -0.6 |
| **POFE1 (45)** | 18.6 | 17.3 | -1.3 |
| **POFE2 (16)** | 64.6 | 55.5 | -9.8 |
| **All POFEs (93)** | 38.6 | 35.0 | -2.3 |
| **POFEs excluding POFE2 (77)** | 33.2 | 30.8 | -0.7 |
| **POFEs without POFE1 and POFE2 (32)** | 53.7 | 52.5 | 0.1 |
| **Night** | | | |
| **Category** | **$RH_{8m}$ (%)** | **$RH_{103m}$ (%)** | **ΔRH (%)** |
| **All data (978)** | 46.3 | 43.0 | -3.2 |
| **POFE1 (1)** | 26.9 | 12.3 | -14.6 |
| **POFE2 (13)** | 50.9 | 53.3 | 2.5 |
| **All POFEs (30)** | 53.0 | 52.1 | -0.1 |
| **POFEs excluding POFE2 (17)** | 54.7 | 51.0 | -2.4 |
| **POFEs without POFE1 and POFE2 (16)** | 56.4 | 53.8 | -1.5 |

***Table S2***

*Mean and SEM of the fluxes of $O_3$ ($FO_3$), NO (FNO) and $NO_2$ ($FNO_2$), downward ozone velocity ($Vdw,O_3$) and ozone mixing ratio ($CO_3$), for the complete dataset, events classified as POFE1, those classified as POFE2, and all POFEs, presented separately during the daytime and nighttime.*

| Data | D/N | Measures | $FO_3$ (nmol $m^{-2}$ $s^{-1}$) | $CO_3$ (ppbv) | $Vdw,O_3$ (cm $s^{-1}$) | FNO (nmol $m^{-2}$ $s^{-1}$) | $FNO_2$ (nmol $m^{-2}$ $s^{-1}$) |
|---|---|---|---|---|---|---|---|
| All data | Day | Mean | -10.94 | 72.46 | 0.39 | 1.77 | 12.49 |
| | | SEM | 0.32 | 0.85 | 0.01 | 0.08 | 0.23 |
| | Night | Mean | -5.07 | 57.91 | 0.33 | 0.32 | 6.79 |
| | | SEM | 0.43 | 0.90 | 0.10 | 0.13 | 0.42 |
| POFE1 | Day | Mean | 4.83 | 76.71 | -0.16 | 1.26 | 11.53 |
| | | SEM | 1.55 | 4.47 | 0.06 | 0.16 | 1.06 |
| | Night | Mean | 0.61 | 36.68 | -0.04 | N/A** | N/A** |
| | | SEM | N/A* | N/A* | N/A* | N/A** | N/A** |
| POFE2 | Day | Mean | 5.24 | 42.88 | -0.29 | -2.17 | -3.77 |
| | | SEM | 2.63 | 5.60 | 0.11 | 0.74 | 1.21 |
| | Night | Mean | 2.87 | 44.69 | -0.18 | -0.88 | -2.42 |
| | | SEM | 1.24 | 5.76 | 0.09 | 0.76 | 1.78 |
| All POFEs | Day | Mean | 4.38 | 69.12 | -0.19 | 0.77 | 9.90 |
| | | SEM | 0.91 | 3.42 | 0.04 | 0.33 | 1.19 |
| | Night | Mean | 2.53 | 52.13 | -0.16 | -0.34 | 3.69 |
| | | SEM | 0.76 | 3.72 | 0.07 | 0.36 | 1.55 |

*SEM could not be calculated because the night bin of POFE1 included only one sample.

**No data available.

## S2. Evidence supporting the role of elevated emission sources in POFE2 occurrence through higher-above measurement NO concentrations (HAMNC) formation

### S2.1 Observational basis for the proposed HAMNC mechanism

Specifically, the analyses show that daytime POFEs become progressively less frequent as the measured net NO flux becomes more positive, whereas the downward $O_3$ velocity generally increases. These relationships indicate that increasingly positive NO fluxes, which are likely associated with a stronger contribution from near-ground NO emissions, generally favor downward $O_3$ fluxes rather than POFE occurrence. Therefore, the persistence of POFE2s even under relatively large positive NO fluxes suggests the influence of an additional process. We propose that the persistence of these events reflects higher-above-measurement NO

concentrations (HAMNC), likely associated with elevated-source NO emissions. Such emissions may continue to reduce or reverse the measured downward $O_3$ flux even when their contribution to the measured net NO flux is partially masked by stronger near-ground NO emissions.

***Table S3***

*Ozone flux ($FO_3$) and downward ozone velocity ($Vdw,O_3$), together with the proportion of positive ozone-flux events (POFEs), shown across NO-flux (FNO) ranges for daytime and nighttime. Values in parentheses refer to the dataset after excluding POFE1s.*

| **FNO (nmol m$^{-2}$ s$^{-1}$) range** | **$FO_3$ (nmol m$^{-2}$ s$^{-1}$)** | **$Vdw,O_3$ (cm s$^{-1}$)** | **POFE proportion** |
|---|---|---|---|
| **Day** | | | |
| **FNO < 0** | -3.99 (-3.87) | 0.09 (0.09) | 0.26 (0.26) |
| **0 ≤ FNO < 0.19** | -6.36 (-6.59) | 0.18 (0.20) | 0.07 (0.03) |
| **0.19 ≤ FNO < 0.90** | -12.37 (-12.47) | 0.32 (0.34) | 0.07 (0.03) |
| **0.90 ≤ FNO < 1.61** | -11.07 (-11.94) | 0.32 (0.36) | 0.05 (0.01) |
| **1.61 ≤ FNO < 2.32** | -9.99 (-10.58) | 0.39 (0.42) | 0.06 (0.02) |
| **2.32 ≤ FNO** | -13.70 (-13.55) | 0.72 (0.73) | 0.03 (0.02) |
| **Night** | | | |
| **FNO < 0** | -3.30 (-3.30) | 0.14 (0.14) | 0.20 (0.20) |
| **0 ≤ FNO < 0.19** | -4.88 (-4.88) | 0.20 (0.20) | 0.13 (0.13) |
| **0.19 ≤ FNO < 0.90** | -7.04 (-7.04) | 0.30 (0.30) | 0.09 (0.09) |
| **0.90 ≤ FNO < 1.61** | -9.52 (-9.52) | 0.70 (0.70) | 0.00 (0.00) |
| **1.61 ≤ FNO < 2.32** | -6.38 (-6.38) | 0.98 (0.98) | 0.00 (0.00) |
| **2.32 ≤ FNO** | -6.54 (-6.54) | 9.95 (9.95) | 0.00 (0.00) |

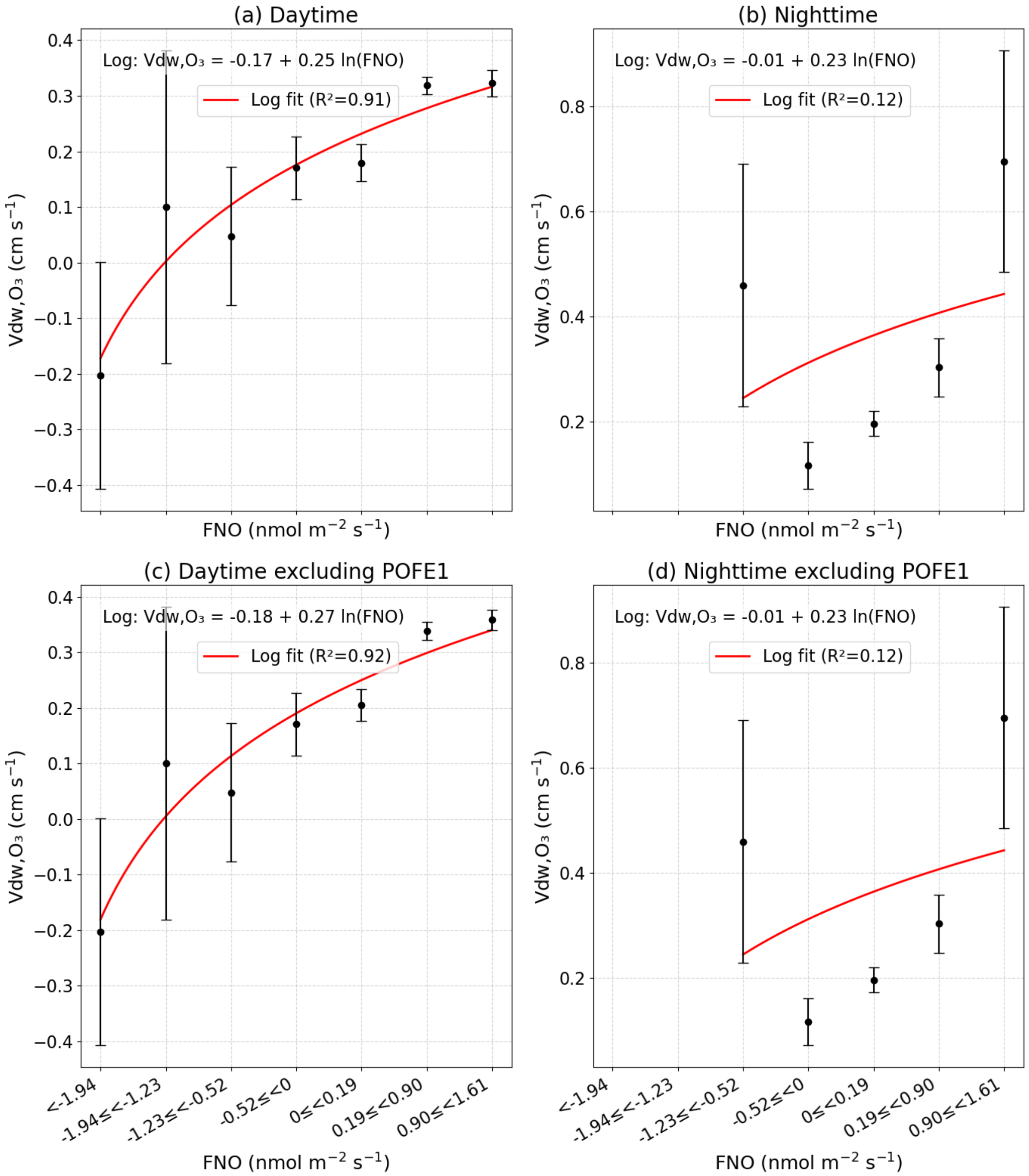


***Fig. S3.*** *Ozone downward velocity (Vdw,$O_3$) vs. NO flux (FNO) focusing on the logarithmic relationship between Vdw,$O_3$ and FNO under low NO emissions (FNO < 1.61 nmol $m^{-2}$ $s^{-1}$). For FNO < 0, data are divided into finer bins of 0.71 nmol $m^{-2}$ $s^{-1}$ (the median absolute deviation of FNO). Black symbols show bin-averaged Vdw,$O_3$ with vertical bars indicating SEM, and red lines show the fitted logarithmic curves. Panels correspond to (a) daytime, (b) nighttime, (c) daytime excluding POFE1s and (d) nighttime excluding POFE1s.*

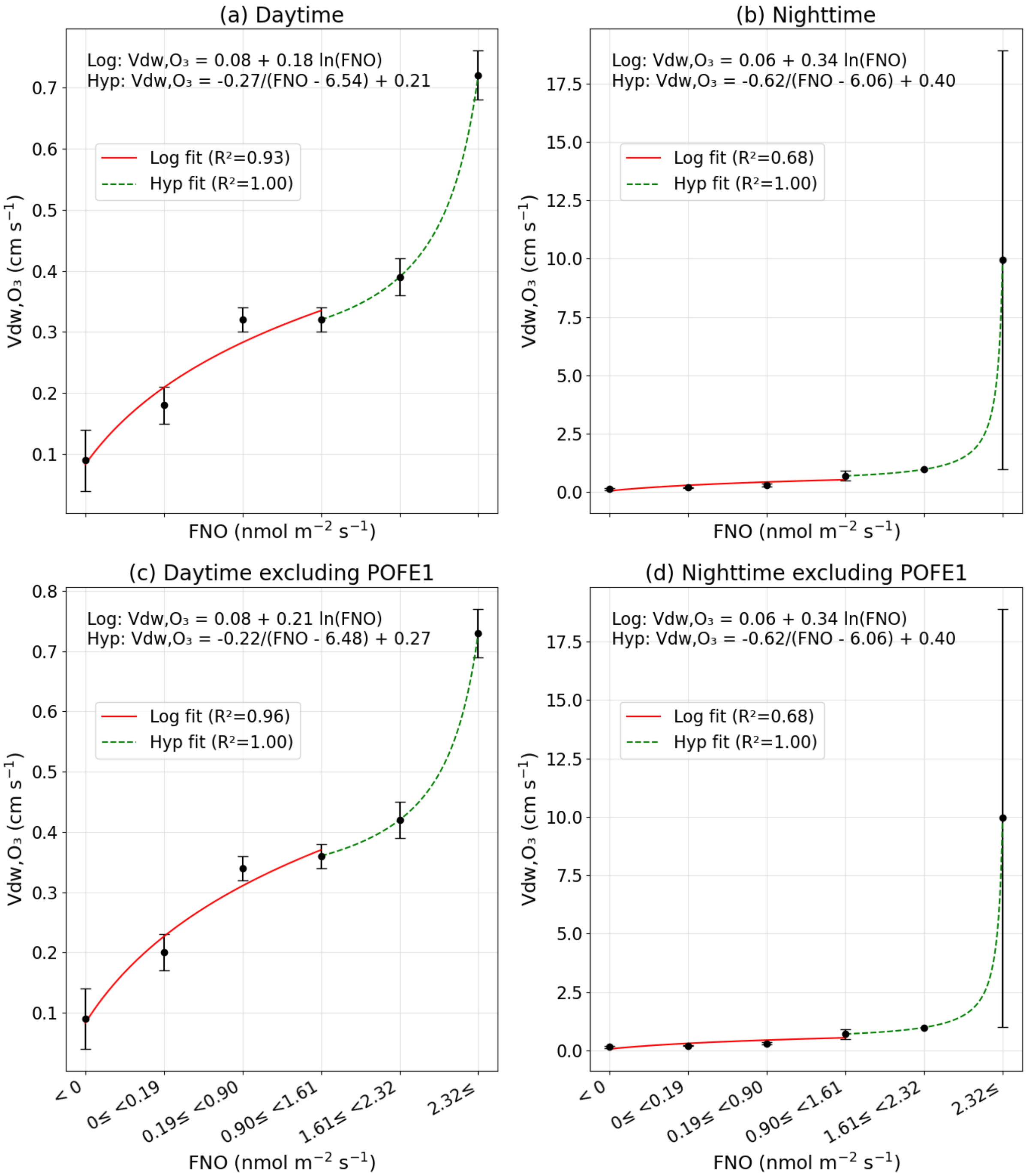


***Fig. S4.*** *Ozone downward velocity (Vdw,$O_3$) vs. NO flux (FNO) range with logarithmic (Log) and hyperbolic (Hyp) fit during (a) daytime, (b) nighttime, (c) daytime without POFE1s and (d) nighttime without POFE1s. Mean values and SEM are shown as filled circles and vertical bars, respectively. Average FNO in the first bin (FNO < 0) is (a) –0.97, (b) –0.31, (c) –0.98, (d) –0.31 nmol* $m^{-2}$ $s^{-1}$.

### S2.2 Potential elevated NO emission sources and supporting evidence for HAMNC formation

Several elevated combustion sources are distributed around the IAP tower and could contribute to HAMNC formation during POFE2 events (see Fig. S5). Most of these sources are located approximately 5–21 km from the measurement site, whereas one source is located 33 km from the IAP tower. These distances are consistent with transport over time scales that can support chemically evolving $NO_x$-rich plumes under suitable atmospheric conditions (e.g., Ryerson et al.[1]; Krol et al.[2]). However, inferring the influence of these sources from the measured net NO flux alone is challenging because strong near-ground traffic emissions can partially mask the contribution of transported $NO_x$-rich plumes to the measured net FNO. The analyses presented below combine wind-direction statistics, traffic patterns, HYSPLIT back trajectories, and a linear regression slope coefficient (LRSC)-based diagnostic to evaluate whether the observations provide consistent support for the proposed HAMNC mechanism.

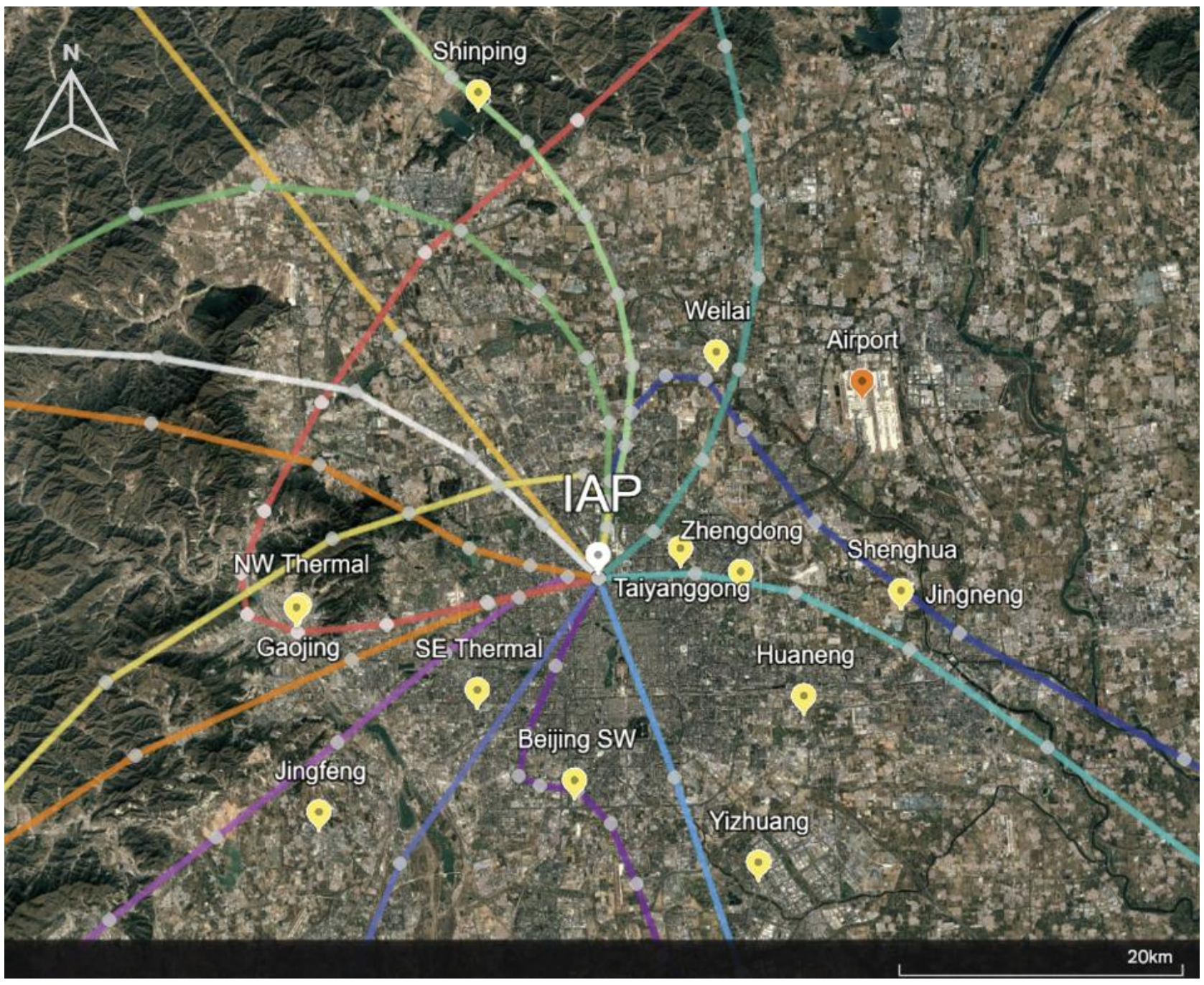


***Fig. S5.*** *HYSPLIT back trajectories ending at 240 m above the IAP tower during periods with FNO < 0 and POFE occurrence, shown together with the major elevated $NO_x$-emitting combustion sources that may contribute to HAMNC formation at the measurement site under suitable transport conditions. The Beijing Capital International Airport is indicated by the orange label. Power-generation facilities (yellow labels) include Huaneng (coal-based, 880 MW; gas-based, 1,921 MW), Gaojing (gas-based, 1,380 MW), Northwest (NW; gas-based, 1,307.8 MW), Shenghua (gas-based, 950.98 MW), Jingneng (gas-based, 845 MW), Southwest (SW; gas-based, 838 MW), Taiyanggong (gas-based, 780 MW), Southeast (SE) Thermal*

*(gas-based, 508 MW), Jingfeng (gas-based, 400 MW), Weilai (gas-based, 255 MW), Yizhuang (gas-based, 150 MW), Zhengdong (gas-based, 122 MW), and Shinping (pumped-storage hydroelectric, 800 MW). Back trajectories were calculated using the NOAA HYSPLIT model with Global Forecast System (GFS) meteorological data and are overlaid on a Google Earth satellite base map. Because HYSPLIT trajectories are available at hourly intervals, trajectories are shown for 16 of the 28 POFE2 cases. Each trajectory is represented by a differently colored line; the remaining 12 cases were excluded because their timestamps did not coincide with the available hourly trajectory output.*

Near-ground traffic emissions, particularly from the major road corridor east of the IAP tower, can generate strong positive NO fluxes and thereby at least partially mask the contribution of elevated NO emissions to the measured net FNO. The masking effect of near-ground emissions is evident from the analysis of the eastern sector, where strongly positive NO-flux events are relatively common. For example, easterly (E) and east-northeasterly (ENE) winds account for 14% and 22%, respectively, of the cases with FNO > 2.32 nmol $m^{-2}$ $s^{-1}$ (Fig. S6). These cases occurred predominantly during the morning traffic-peak hours, indicating that strong near-ground traffic emissions (Fig. S7) from the Beijing–Tibet Expressway, which runs approximately north–south and is located 330 m east of the IAP tower (Fig. S8), likely dominated the measured net FNO. The strong near-ground traffic emissions from this road can shift the measured net FNO towards positive values, making the contribution of NO transported from the eastern sector to the measured net FNO less apparent (Fig. S6). Accordingly, although several elevated combustion sources are located east of the measurement site, including the Taiyanggong Power Plant (39°58′42″N, 116°26′19″E) and the Zhendong Power Plant (39°57′49″N, 116°29′16″E) (Fig. S5), negative NO fluxes were observed less frequently under easterly winds.

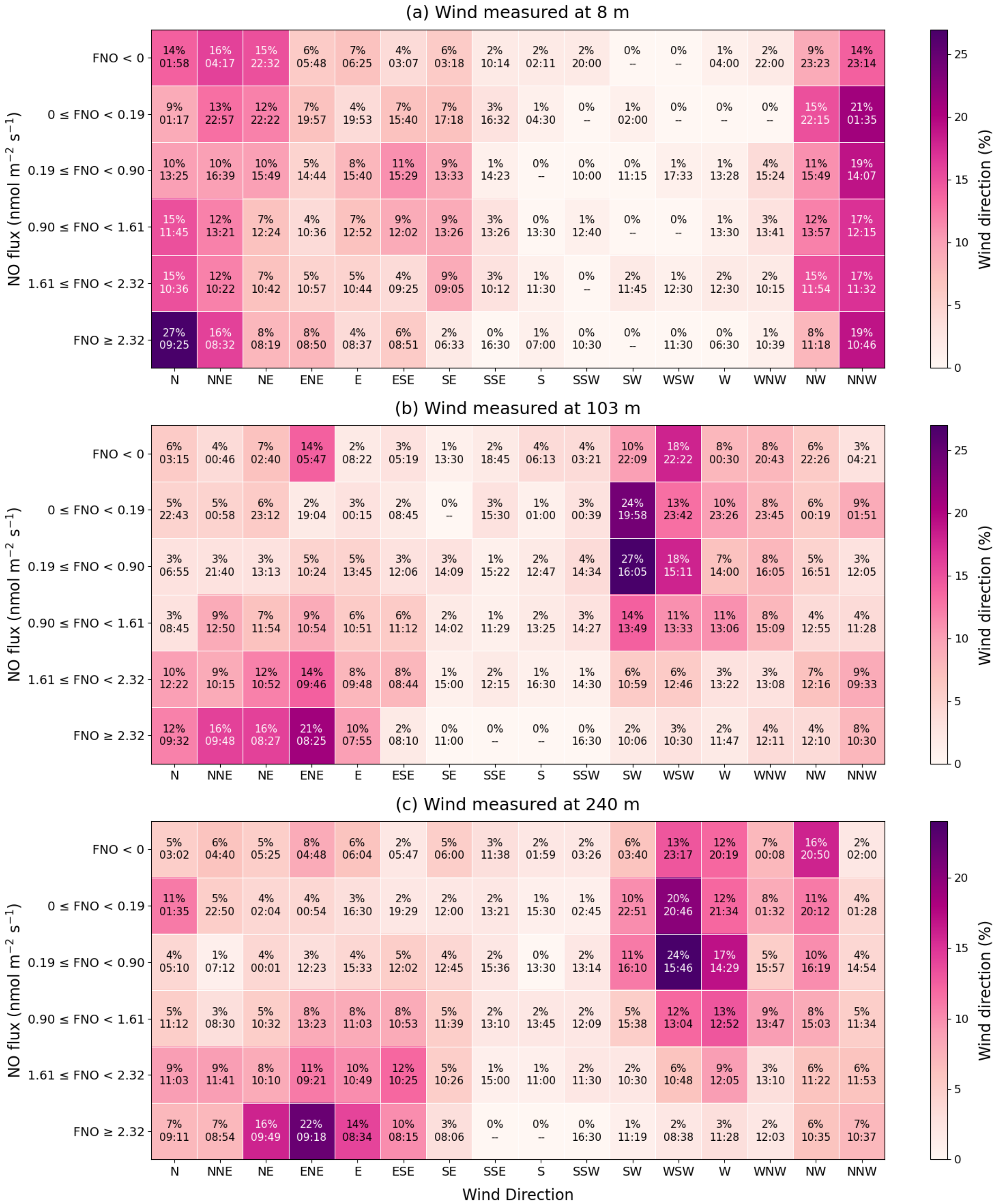


***Fig. S6.*** *Wind-direction frequency (%) and the mean local hour for different NO-flux ranges at three measurement heights, using 16 wind-direction sectors. For each NO-flux bin, upper values in the cells indicate the percentage of observations from each wind sector (rows sum to 100%), lower values indicate the mean local hour, and color shading is proportional to the corresponding frequency. Panels show wind directions measured at (a) 8 m (near ground), (b) 103 m (closest measurement height to the eddy-covariance system; EC height: 102 m), and (c) 240 m above ground level.*

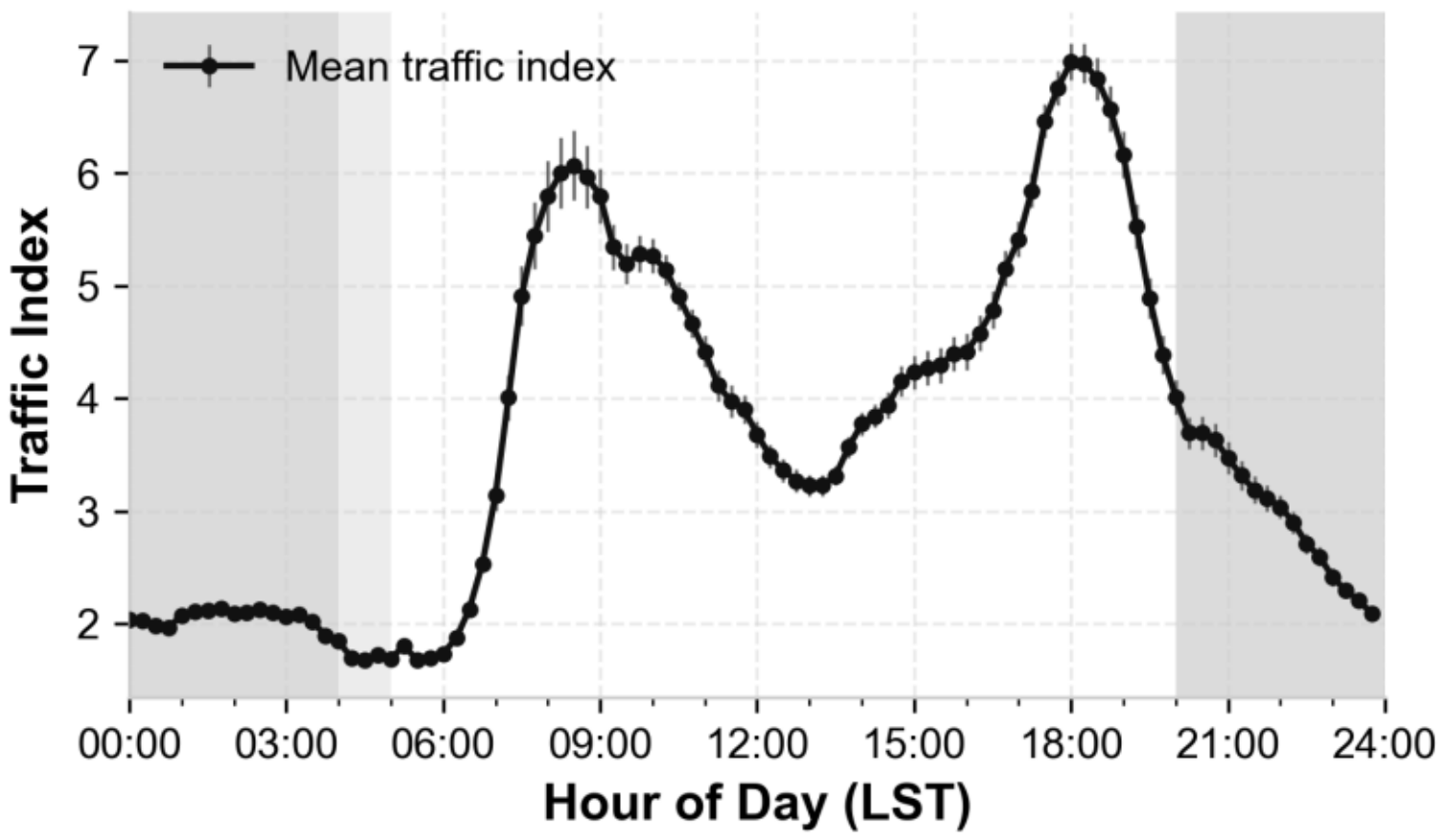


***Fig. S7.*** *Diurnal profile of Beijing Traffic Index during the measurement periods (2 May–25 June 2023). The Beijing Traffic Index, ranging from 0-10, indicates the traffic condition from unimpeded flow to severe congestion. The darker and lighter shaded areas represent, respectively, nighttime for the entire measurement period, and nighttime for part of the measurement period, considering changes in daylight hours during the observation period. Filled circles and vertical bars represent averages and standard errors of the mean, respectively.*

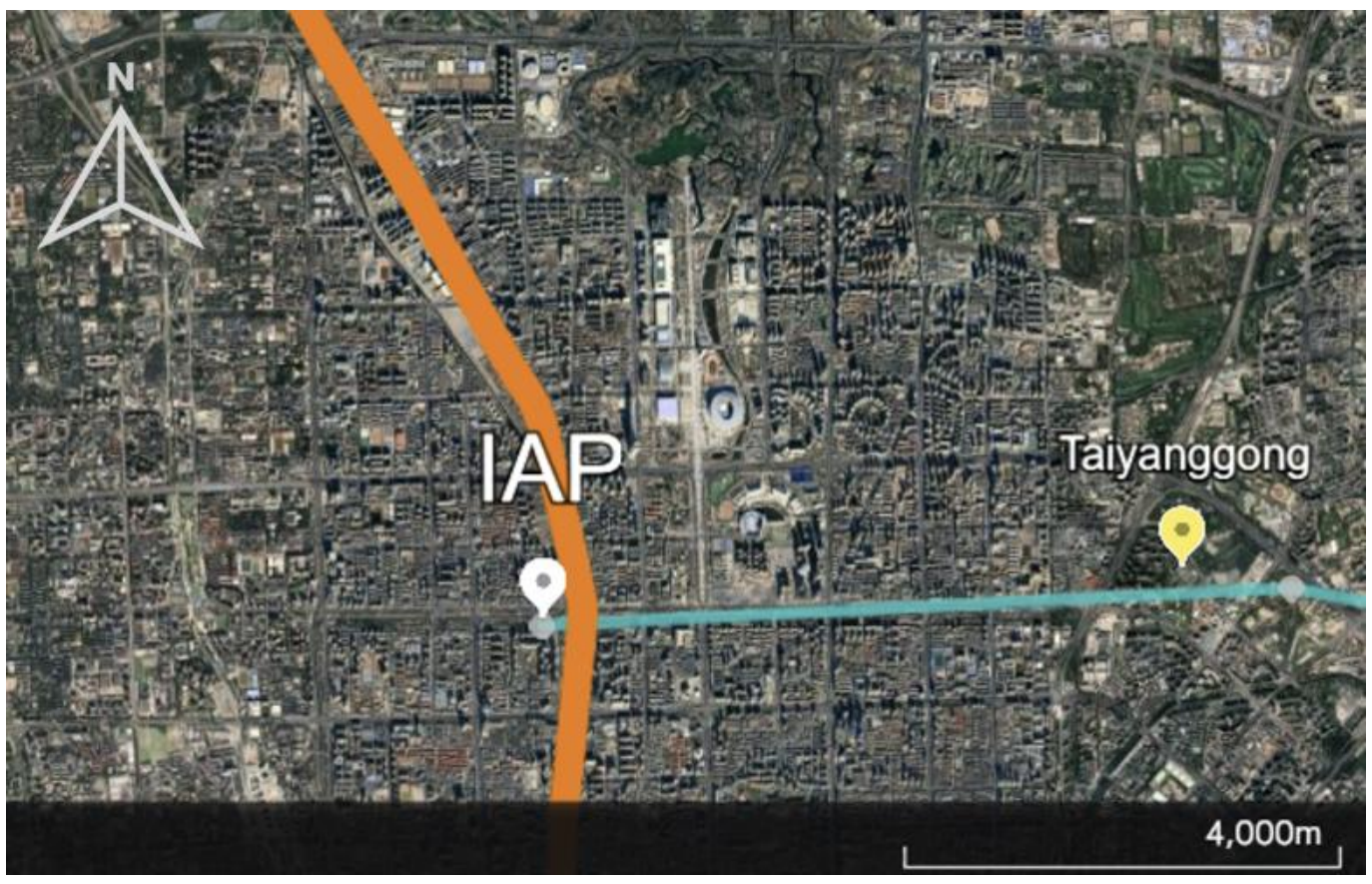


***Fig. S8.*** *HYSPLIT back trajectory initialized at 240 m above the IAP tower. Thin teal line indicates the back trajectory, and the thick orange line indicates the Beijing–Tibet Expressway east of the IAP site. The representative case on 11 June 2023 at 06:00 local time shows an easterly transport pathway passing near the Taiyanggong Power Plant, suggesting that the elevated sources east of IAP site may have contributed to negative FNO and POFE occurrence when the traffic-related near-ground NO contribution is relatively weak.*

In contrast, during nighttime negative FNO events did occur under E and ENE winds, accounting for 6% and 8% of the cases at 240 m and 2% and 14% of the cases at 103 m, respectively (Fig. S6). These events occurred predominantly during the night and early morning (approximately 02:30–06:00 local time), when traffic emissions were expected to be weaker (see Fig. S7), supporting the interpretation that elevated NO sources from the eastern sector can

contribute to negative FNO when the competing influence of near-ground traffic emissions is reduced.

To further evaluate this interpretation, we performed HYSPLIT back-trajectory analysis initialized at 240 m above ground level for periods associated with FNO < 0 and POFE occurrence (Fig. S5). One representative case (11 June 2023 at 06:00 local time) exhibited an easterly transport pathway passing through the vicinity of the Taiyanggong Power Plant (39°58′42″N, 116°26′19″E), located approximately 5 km east of the IAP site (Fig. S8), before the morning traffic peak occurred (Fig. S7). Although this represents only a single illustrative case rather than a statistical attribution, it demonstrates that transport from elevated combustion sources located east of the tower is physically plausible during periods associated with negative FNO and POFE occurrence.

To provide a more complete assessment, we performed an additional diagnostic analysis based on the linear regression slope coefficient (LRSC) between Vdw,$O_3$ and NO concentration across different FNO classes and wind-direction sectors. Negative LRSC values are associated with conditions under which increasing NO concentrations are associated with decreasing Vdw,$O_3$, consistent with relatively stronger $O_3$ titration occurring above the EC measurement height than at the EC measurement height. Note that such conditions may result in HAMNC, but not necessarily. To ensure sufficient statistical robustness, each LRSC was calculated using at least five data points. We then calculated $\chi$ as the percentage contribution of the absolute magnitude of negative LRSCs to the total absolute magnitude of all LRSCs within each wind direction sector and NO bin as follows:

$$\chi(\%) = \frac{\sum_{i=1}^{n} |\mathrm{LRSC}(Vdw, O_3, C_{NO})_{negative}(i)|}{\sum_{i=1}^{o} |\mathrm{LRSC}(Vdw, O_3, C_{NO})\ (i)|} \times 100$$

where $\mathrm{LRSC}(\mathrm{Vdw,O_3}, \mathrm{C_{NO}})_{\mathrm{negative}}(i)$ and $\mathrm{LRSC}(\mathrm{Vdw,O_3}, \mathrm{C_{NO}})\ (i)$ represent the LRSC values for the regression between Vdw,$O_3$ and $\mathrm{C_{NO}}$ when it is negative, and when it is either negative or positive, respectively. Here, $n$ and $o$ are the number of LRSC estimates associated with these two categories; negative and total cases, respectively.

Figure S9 shows that, under FNO < 0, high $\chi$ values occur mainly in the SW and NW sectors, indicating that negative LRSCs dominated the total LRSC magnitude in these sectors. In the 16-sector classification used in Fig. S1, these broad sectors correspond approximately to the SSW–WSW and WNW–NNW directions, respectively, where negative FNO events were frequently observed. This suggests that westerly to northwesterly flow was associated not only

with frequent downward NO fluxes but also with a strong negative sensitivity of $V_{dw,O_3}$ to $C_{NO}$. Furthermore, elevated $\chi$ values are also observed in the northeastern sector, despite the lower occurrence frequency of negative FNO in this direction (Fig. S1). This indicates that, although negative FNO events were less frequent under northeasterly flow, negative LRSCs still dominated the LRSC magnitude among the available LRSC estimates. This northeastern signal may reflect the influence of emissions from the Weilai Power Plant (40°06′05″N, 116°28′05″E), located approximately 16 km from the IAP site (Fig. S5). In addition, Beijing Capital International Airport (40°04′51″N, 116°34′25″E), located about 21 km northeast of the IAP site, may also contribute to elevated $NO_x$ concentrations under suitable transport conditions and therefore represents another potential contributor to HAMNC formation.

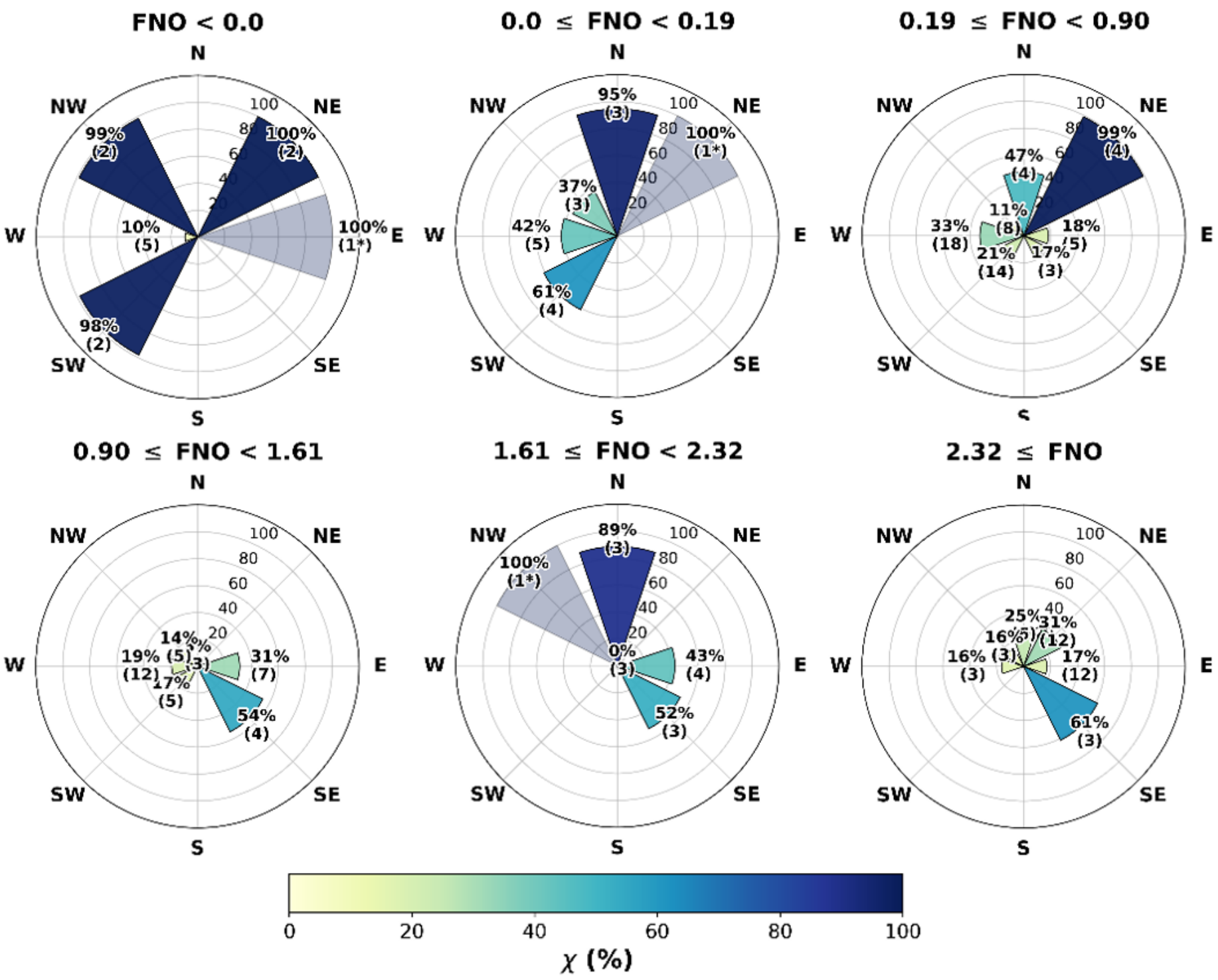


***Fig. S9.*** *LRSC-based diagnostic analysis for different FNO bins (nmol m⁻² s⁻¹). Color shading indicates χ (%), defined as the percentage contribution of the summed absolute magnitudes of negative LRSC estimates to the summed absolute magnitudes of all LRSC estimates within each wind-direction sector and FNO bin. Numbers in parentheses indicate the number of calculated LRSC estimates (o) in each sector. Pale colors denote sectors with fewer than two LRSC estimates (o < 2), which have limited sample support and should therefore be interpreted qualitatively only. For clarity, χ is shown using eight broad wind-direction sectors, whereas the occurrence-frequency heatmap in Fig. S1 is based on 16 wind-direction sectors.*

Consistent with the findings of Li et al.,[3] the presence of elevated $\chi$ values outside the dominant negative-FNO sectors suggests that the influence of NO emissions from elevated sources on the measured net FNO is not confined to those sectors. This further supports our interpretation that near-ground traffic emissions can partially mask the contribution of NO emissions from elevated sources to the measured net NO flux. Thus, the influence of elevated NO sources cannot be inferred solely from the occurrence of negative FNO events or from a single transport direction. Notably, even under $\mathrm{FNO} > 2.32$ nmol m$^{-2}$ s$^{-1}$, where near-ground traffic emissions are expected to dominate the measured net NO flux, relatively high $\chi$ values remain evident in the southeastern sector. Moreover, two POFE2 events were identified in the southeastern sector during periods of negligible near-ground traffic (23:00 and 04:00; Fig. S7). Thus, the influence of elevated-source NO emissions cannot be identified solely from the sign or magnitude of the measured net FNO. Instead, the combined evidence from wind-direction analysis, HYSPLIT back trajectories, and the LRSC diagnostic suggests that elevated NO sources can affect $O_3$ deposition even when their contribution to the measured net FNO is partially masked by near-ground traffic emissions. Collectively, the wind-direction analysis, HYSPLIT back trajectories, and LRSC diagnostic support the interpretation that HAMNC, likely associated with NO emissions from elevated sources, can contribute to reductions in the observed downward $O_3$ fluxes, while its effect on the measured net FNO is partially masked by stronger near-ground traffic emissions.

## S3. Effect of air dryness on $O_3$ dry deposition

Table S4 summarizes the occurrence and proportion of POFEs during our measurements, separately for day and night.

***Table S4***

***(a)*** *Ozone flux ($FO_3$) and downward velocity ($V_{dw,O_3}$) with positive ozone-flux event (POFE) proportion by relative humidity (RH) range during the daytime and nighttime. Fisher's exact test was applied to evaluate whether the occurrence frequency of POFEs within each RH bin differs significantly from that in the remaining data. The resulting p-values (in parentheses) indicate the statistical significance of POFE occurrences in each RH category. The number of data points (n) in each RH range represents the total count of valid 30-min flux data used in the analysis.*

| **Day** | | | | | | |
|---|---|---|---|---|---|---|
| **RH range (n)**<br>**$FO_3$ characteristics** | **RH ≥ 30% (647)** | **RH < 30% (455)** | **25 ≤ RH < 30% (99)** | **20 ≤ RH < 25% (135)** | **10 ≤ RH < 20% (215)** | **RH < 10% (6)** |
| **POFE proportion (*p*-value)** | 0.07 (0.154) | 0.10 (0.078) | 0.09 (0.705) | 0.07 (0.868) | 0.11 (0.099) | 0.50 (0.009) |
| **$V_{dw,O_3}$ ($cm\ s^{-1}$)** | 0.45 | 0.31 | 0.36 | 0.33 | 0.28 | 0.00 |
| **$FO_3$ ($nmol\ m^{-2}\ s^{-1}$)** | -11.59 | -10.22 | -12.36 | -10.55 | -9.31 | 0.07 |
| **Night** | | | | | | |
| **RH range (n)**<br>**$FO_3$ characteristics** | **RH ≥ 30% (172)** | **RH < 30% (13)** | **25 ≤ RH < 30% (9)** | **20 ≤ RH < 25% (4)** | **10 ≤ RH < 20% (0)** | **RH < 10% (0)** |
| **POFE proportion (*p*-value)** | 0.17 (0.472) | 0.08 (0.696) | 0.11 (1.000) | 0.00 (1.000) | N/A (-) | N/A (-) |
| **$V_{dw,O_3}$ ($cm\ s^{-1}$)** | 0.33 | 0.25 | 0.25 | 0.24 | N/A | N/A |
| **$FO_3$ ($nmol\ m^{-2}\ s^{-1}$)** | -5.09 | -5.12 | -5.96 | -3.24 | N/A | N/A |

***(b)*** *$FO_3$ and $V_{dw,O_3}$ with POFE proportion by RH range during the daytime and nighttime, excluding POFE observations associated with negative NO flux (POFE2s).*

| **Day** | | | | | | |
|---|---|---|---|---|---|---|
| **RH range (n)**<br>**$FO_3$ characteristics** | **RH ≥ 30% (632)** | **RH < 30% (454)** | **25 ≤ RH < 30% (98)** | **20 ≤ RH < 25% (135)** | **10 ≤ RH < 20% (215)** | **RH < 10% (6)** |
| **POFE proportion (*p*-value)** | 0.05 (0.004) | 0.10 (0.002) | 0.08 (0.676) | 0.07 (0.857) | 0.11 (0.011) | 0.50 (0.006) |
| **$V_{dw,O_3}$ (cm $s^{-1}$)** | 0.47 | 0.31 | 0.36 | 0.33 | 0.28 | 0.00 |
| **$FO_3$ (nmol $m^{-2}$ $s^{-1}$)** | -11.99 | -10.26 | -12.58 | -10.55 | -9.31 | 0.07 |
| **Night** | | | | | | |
| **RH range (n)**<br>**$FO_3$ characteristics** | **RH ≥ 30% (159)** | **RH < 30% (13)** | **25 ≤ RH < 30% (9)** | **20 ≤ RH < 25% (4)** | **10 ≤ RH < 20% (0)** | **RH < 10% (0)** |
| **POFE proportion (*p*-value)** | 0.10 (1.000) | 0.07 (1.000) | 0.11 (1.000) | 0.00 (1.000) | N/A (-) | N/A (-) |
| **$V_{dw,O_3}$ (cm $s^{-1}$)** | 0.37 | 0.25 | 0.25 | 0.24 | N/A | N/A |
| **$FO_3$ (nmol $m^{-2}$ $s^{-1}$)** | -5.74 | -5.12 | -5.96 | -3.24 | N/A | N/A |

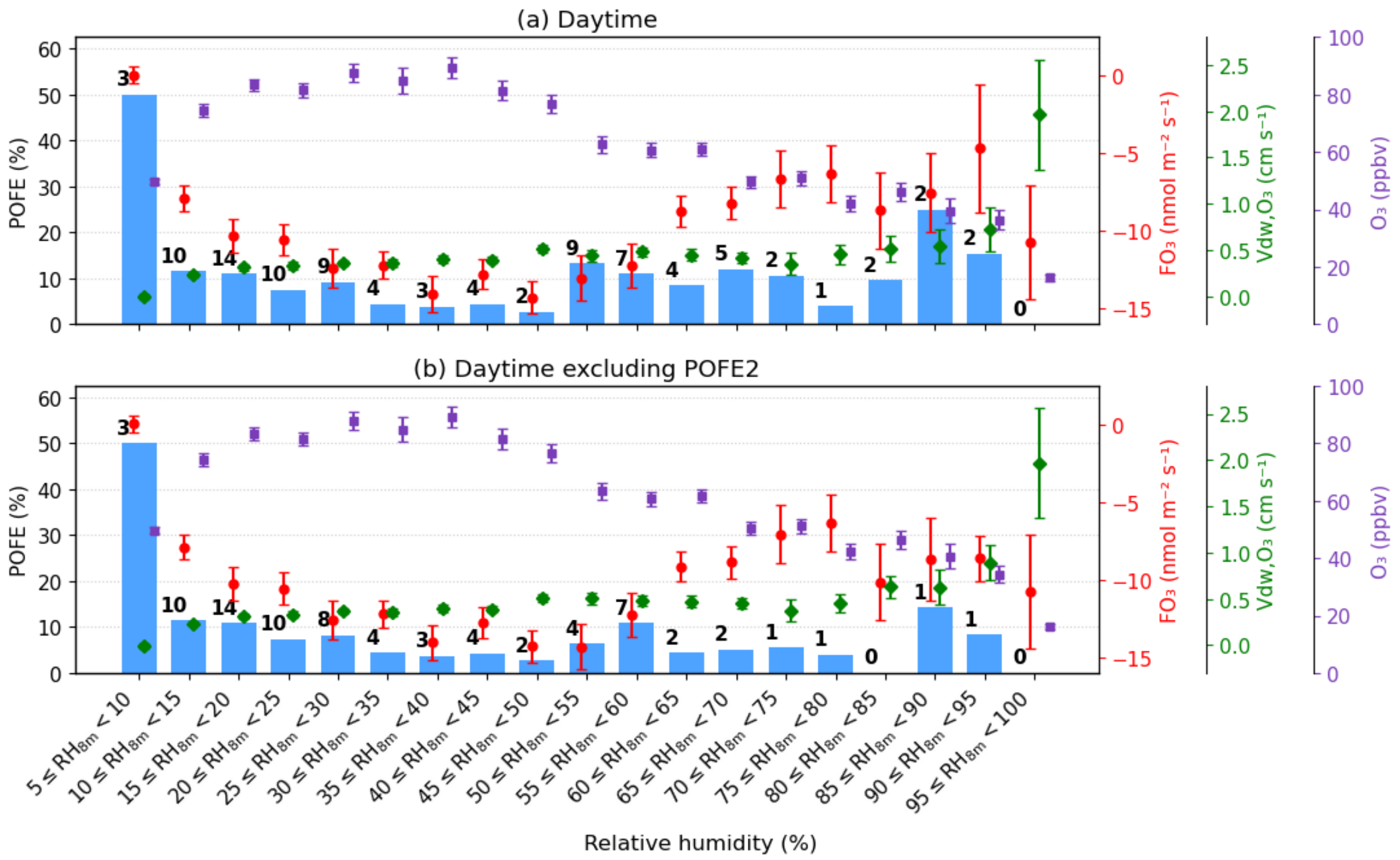


***Fig. S10.*** *Ozone flux ($FO_3$; red), mixing ratio (purple) and downward velocity ($V_{dw,O_3}$; green) plotted with the proportion of positive ozone-flux events (POFEs; blue bars) across relative humidity (RH) bins for (a) daytime and (b) daytime excluding POFE2s. Numbers above the blue bars indicate the count of POFEs in each RH bin. Filled circles, squares and diamonds represent mean values, and vertical bars indicate SEM.*

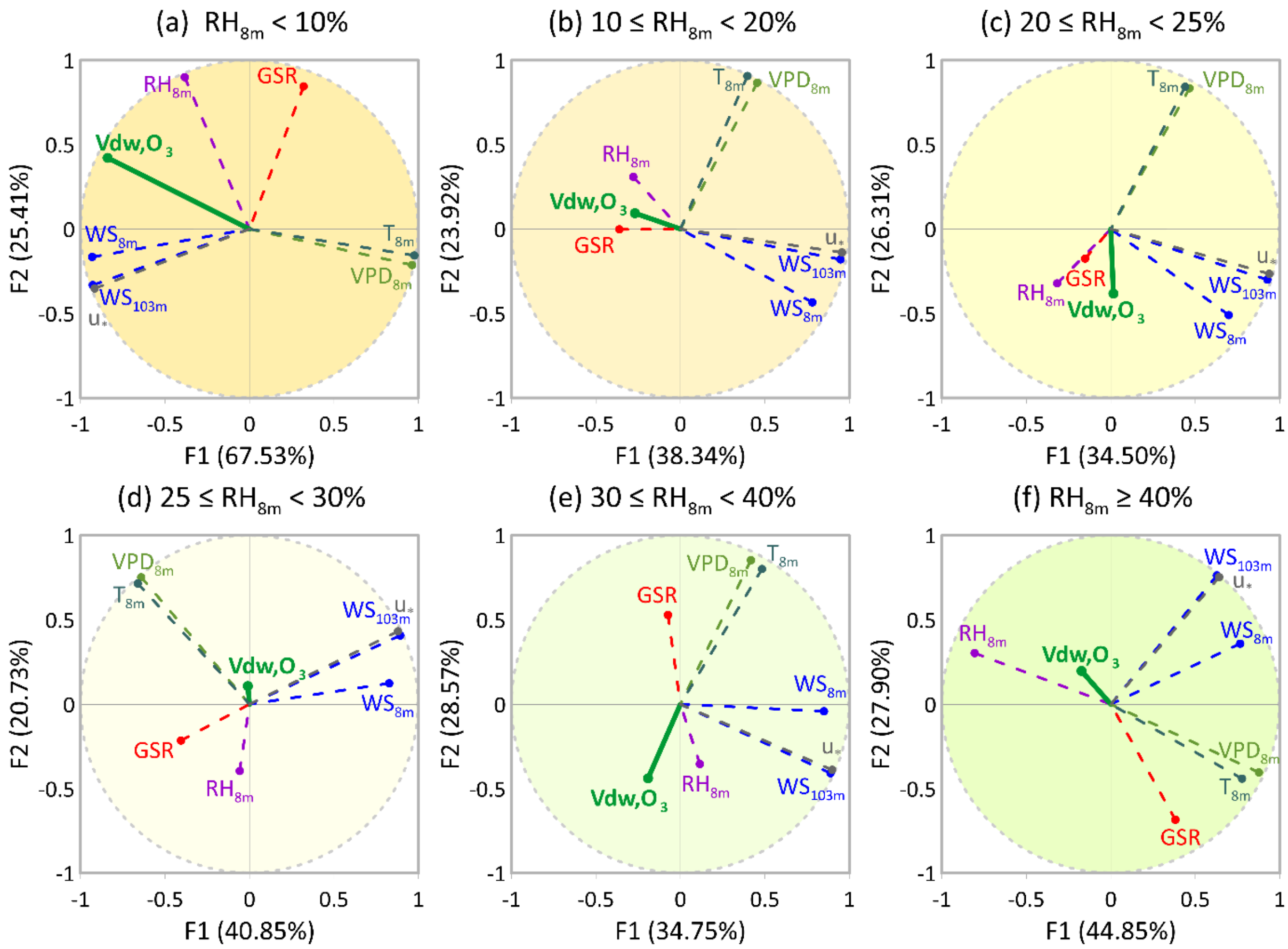


***Fig. S11.*** *PCA results for daytime conditions (defined as periods with positive global solar radiation) based on half-hourly observations across relative humidity (RH) ranges, excluding type-2 positive ozone-flux events (POFE2s) (same as Fig. 4 in the main text, but with POFE2 removed). (a) $RH_{8m}$ < 10%, (b) 10% ≤ $RH_{8m}$ < 20%, (c) 20% ≤ $RH_{8m}$ < 25%, (d) 25% ≤ $RH_{8m}$ < 30%, (e) 30% ≤ $RH_{8m}$ < 40% and (f) $RH_{8m}$ ≥ 40%. Abbreviations: Vdw,$O_3$ (downward velocity of ozone), RH (relative humidity), GSR (global solar radiation), T (temperature), VPD (vapor-pressure deficit), WS (wind speed) and $u_*$ (friction velocity). See Sect. 2.1 for details on the elevation indexing of meteorological parameters.*

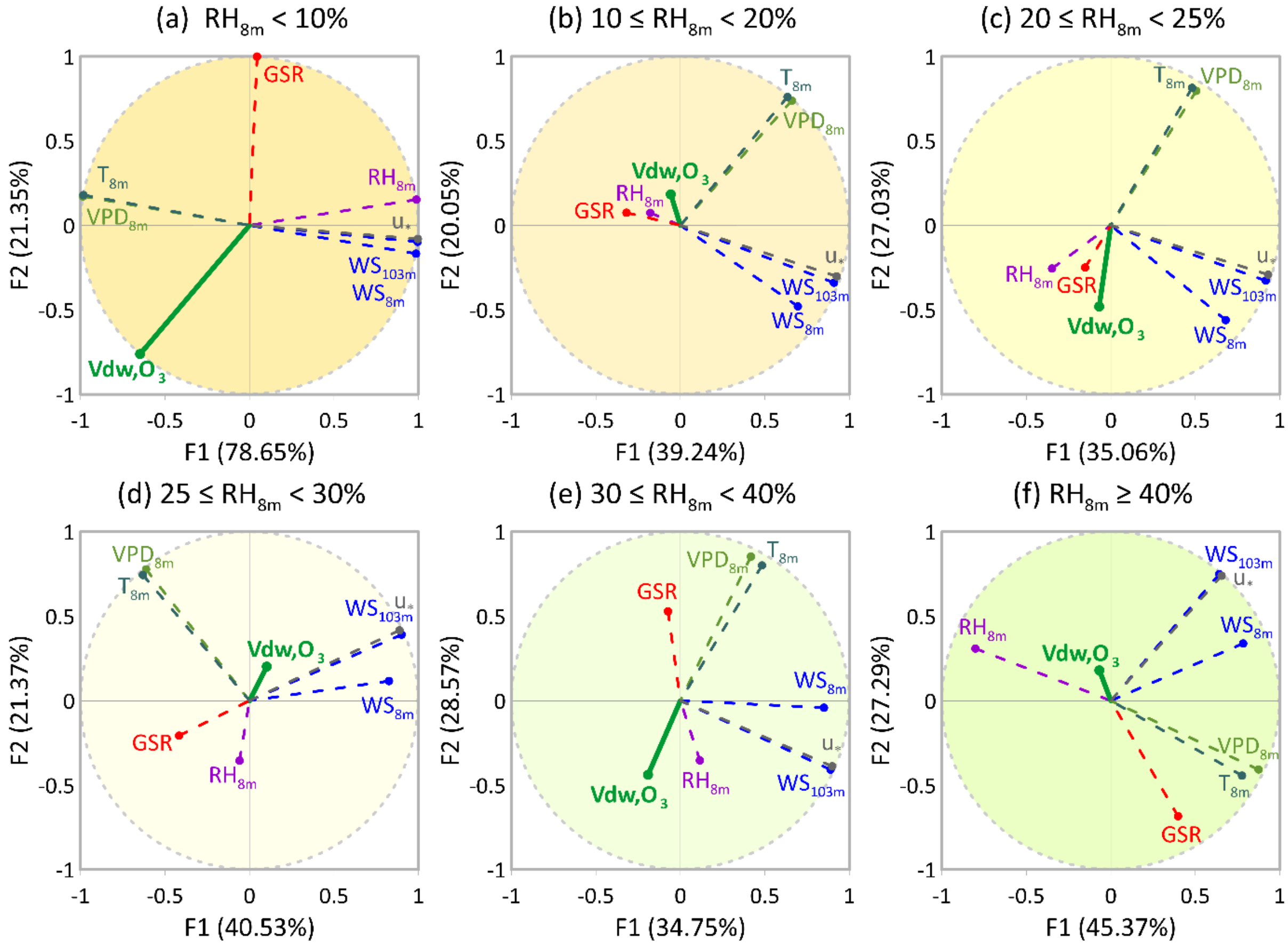


***Fig. S12.*** *PCA results of daytime conditions (defined as periods with positive global solar radiation) based on half-hourly observations across relative humidity (RH) ranges excluding type-1 positive ozone-flux events (POFE1s) (same as Fig. 4 in the main text, but with POFE1 removed). (a)* $RH_{8m} < 10\%$*, (b)* $10\% \leq RH_{8m} < 20\%$*, (c)* $20\% \leq RH_{8m} < 25\%$*, (d)* $25\% \leq RH_{8m} < 30\%$*, (e)* $30\% \leq RH_{8m} < 40\%$ *and (f)* $RH_{8m} \geq 40\%$*. Abbreviations:* $Vdw,O_3$ *(downward velocity of ozone), RH (relative humidity), GSR (global solar radiation), T (temperature), VPD (vapor-pressure deficit), WS (wind speed) and* $u_*$ *(friction velocity). See Sect. 2.1 for details on meteorological parameters elevation indexing.*

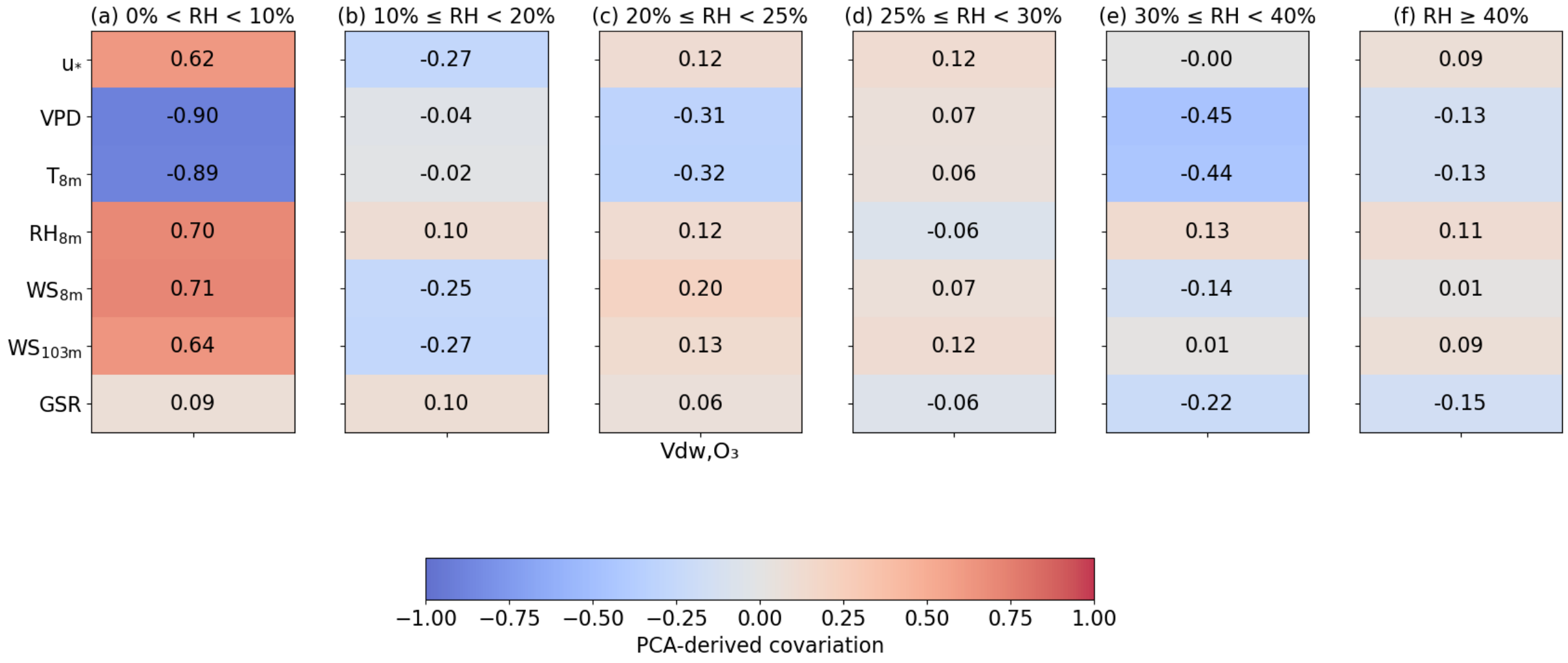


| | (a) 0% < RH < 10% | (b) 10% ≤ RH < 20% | (c) 20% ≤ RH < 25% | (d) 25% ≤ RH < 30% | (e) 30% ≤ RH < 40% | (f) RH ≥ 40% |
|---|---|---|---|---|---|---|
| $u_*$ | 0.62 | -0.27 | 0.12 | 0.12 | -0.00 | 0.09 |
| VPD | -0.90 | -0.04 | -0.31 | 0.07 | -0.45 | -0.13 |
| $T_{8m}$ | -0.89 | -0.02 | -0.32 | 0.06 | -0.44 | -0.13 |
| $RH_{8m}$ | 0.70 | 0.10 | 0.12 | -0.06 | 0.13 | 0.11 |
| $WS_{8m}$ | 0.71 | -0.25 | 0.20 | 0.07 | -0.14 | 0.01 |
| $WS_{103m}$ | 0.64 | -0.27 | 0.13 | 0.12 | 0.01 | 0.09 |
| GSR | 0.09 | 0.10 | 0.06 | -0.06 | -0.22 | -0.15 |

***Fig. S13.*** *PCA-derived covariation coefficients between downward ozone velocity (Vdw,$O_3$) and meteorological variables—wind speed (WS), temperature (T), vapor-pressure deficit (VPD), global solar radiation (GSR) and the friction velocity ($u_*$)—across bin-based relative humidity ($RH_{8m}$) classes for the same PCA presented in Fig. 4 of the main text. Coefficients were derived from the factor loadings in Fig. 4 as the sum of the products of the loadings of Vdw,$O_3$ and each meteorological variable on the first two principal components (F1 and F2), and range from −1 to 1 as indicated by the color bar (blue = negative, red = positive). Results are shown for (a) $RH_{8m}$ < 10%, (b) 10% ≤ $RH_{8m}$ < 20%, (c) 20% ≤ $RH_{8m}$ < 25%, (d) 25% ≤ $RH_{8m}$ < 30%, (e) 30% ≤ $RH_{8m}$ < 40% and (f) $RH_{8m}$ ≥ 40%. See Sect. 2.1 for details on meteorological parameters elevation indexing.*

***Table S5***

***(a)*** *Factor loadings from the PCA performed for daytime conditions under relative humidity (RH) < 10%. PCA visualization of full dataset is shown in Fig. 4a of the main text; corresponding analyses excluding POFE1s and POFE2s are shown in Figs. S12a and S11a, respectively. Abbreviations:* $V_{dw,O_3}$ *(downward velocity of ozone), GSR (global solar radiation), T (temperature), VPD (vapor-pressure deficit), WS (wind speed) and* $u_*$ *(friction velocity). See Sect. 2.1 for details on meteorological parameters elevation indexing.*

| **Variable** | **Whole data** | | **Excluding POFE1** | | **Excluding POFE2** | |
|---|---|---|---|---|---|---|
| | **F1 (67.53%)** | **F2 (25.41%)** | **F1 (78.65%)** | **F2 (21.35%)** | **F1 (67.53%)** | **F2 (25.41%)** |
| **$V_{dw,O_3}$** | -0.84 | 0.42 | -0.65 | -0.76 | -0.84 | 0.42 |
| **$u_*$** | -0.92 | -0.35 | 1.00 | -0.08 | -0.92 | -0.35 |
| **$VPD_{8m}$** | 0.96 | -0.21 | -0.99 | 0.17 | 0.96 | -0.21 |
| **$T_{8m}$** | 0.98 | -0.16 | -0.98 | 0.18 | 0.98 | -0.16 |
| **$RH_{8m}$** | -0.38 | 0.90 | 0.99 | 0.15 | -0.38 | 0.90 |
| **$WS_{8m}$** | -0.93 | -0.17 | 0.99 | -0.17 | -0.93 | -0.17 |
| **$WS_{103m}$** | -0.93 | -0.33 | 1.00 | -0.10 | -0.93 | -0.33 |
| **GSR** | 0.32 | 0.85 | 0.05 | 1.00 | 0.32 | 0.85 |

***(b)*** *Factor loadings from the PCA performed for daytime conditions under 10% ≤ RH < 20%. PCA visualization of full dataset is shown in Fig. 4b of the main text; corresponding analyses excluding POFE1s and POFE2s are shown in Figs. S12b and S11b, respectively. See Sect. 2.1 for details on meteorological parameters elevation indexing.*

| **Variable** | **Whole data** | | **Excluding POFE1** | | **Excluding POFE2** | |
|---|---|---|---|---|---|---|
| | **F1 (38.34%)** | **F2 (23.92%)** | **F1 (39.24%)** | **F2 (20.05%)** | **F1 (38.34%)** | **F2 (23.92%)** |
| **$V_{dw,O_3}$** | -0.27 | 0.09 | -0.06 | 0.18 | -0.27 | 0.09 |
| **$u_*$** | 0.96 | -0.14 | 0.93 | -0.30 | 0.96 | -0.14 |
| **$VPD_{8m}$** | 0.45 | 0.87 | 0.66 | 0.74 | 0.45 | 0.87 |
| **$T_{8m}$** | 0.40 | 0.91 | 0.63 | 0.76 | 0.40 | 0.91 |
| **$RH_{8m}$** | -0.28 | 0.31 | -0.18 | 0.07 | -0.28 | 0.31 |
| **$WS_{8m}$** | 0.78 | -0.43 | 0.70 | -0.48 | 0.78 | -0.43 |
| **$WS_{103m}$** | 0.95 | -0.18 | 0.91 | -0.34 | 0.95 | -0.18 |
| **GSR** | -0.36 | 0.00 | -0.32 | 0.07 | -0.36 | 0.00 |

***(c)*** *Factor loadings from the PCA performed for daytime conditions under 20% ≤ RH < 25%. PCA visualization of full dataset is shown in Fig. 4c of the main text; corresponding analyses excluding POFE1s and POFE2s are shown in Figs. S12c and S11c, respectively. See Sect. 2.1 for details on meteorological parameters elevation indexing.*

| Variable | Whole data | | Excluding POFE1 | | Excluding POFE2 | |
|---|---|---|---|---|---|---|
| | F1 (34.50%) | F2 (26.31%) | F1 (35.06%) | F2 (27.03%) | F1 (34.50%) | F2 (26.31%) |
| $V_{dw,O_3}$ | 0.01 | -0.38 | -0.07 | -0.48 | 0.01 | -0.38 |
| $u_*$ | 0.94 | -0.27 | 0.93 | -0.29 | 0.94 | -0.27 |
| $VPD_{8m}$ | 0.47 | 0.83 | 0.51 | 0.80 | 0.47 | 0.83 |
| $T_{8m}$ | 0.44 | 0.84 | 0.48 | 0.81 | 0.44 | 0.84 |
| $RH_{8m}$ | -0.32 | -0.32 | -0.35 | -0.26 | -0.32 | -0.32 |
| $WS_{8m}$ | 0.70 | -0.51 | 0.68 | -0.56 | 0.70 | -0.51 |
| $WS_{103m}$ | 0.93 | -0.30 | 0.92 | -0.33 | 0.93 | -0.30 |
| GSR | -0.15 | -0.17 | -0.15 | -0.25 | -0.15 | -0.17 |

***(d)*** *Factor loadings from the PCA performed for daytime conditions under 25% ≤ RH < 30%. PCA visualization of full dataset is shown in Fig. 4d of the main text; corresponding analyses excluding POFE1s and POFE2s are shown in Figs. S12d and S11d, respectively. See Sect. 2.1 for details on meteorological parameters elevation indexing.*

| Variable | Whole data | | Excluding POFE1 | | Excluding POFE2 | |
|---|---|---|---|---|---|---|
| | F1 (41.36%) | F2 (20.57%) | F1 (40.53%) | F2 (21.37%) | F1 (40.85%) | F2 (20.73%) |
| $V_{dw,O_3}$ | 0.06 | 0.15 | 0.10 | 0.20 | -0.01 | 0.11 |
| $u_*$ | 0.88 | 0.42 | 0.89 | 0.42 | 0.88 | 0.43 |
| $VPD_{8m}$ | -0.64 | 0.75 | -0.61 | 0.78 | -0.64 | 0.75 |
| $T_{8m}$ | -0.67 | 0.71 | -0.63 | 0.74 | -0.66 | 0.71 |
| $RH_{8m}$ | -0.08 | -0.40 | -0.06 | -0.36 | -0.06 | -0.40 |
| $WS_{8m}$ | 0.83 | 0.12 | 0.83 | 0.12 | 0.83 | 0.12 |
| $WS_{103m}$ | 0.90 | 0.40 | 0.90 | 0.39 | 0.89 | 0.41 |
| GSR | -0.41 | -0.21 | -0.42 | -0.21 | -0.41 | -0.21 |

***(e)*** *Factor loadings from the PCA performed for daytime conditions under 30% ≤ RH < 40%. PCA visualization of full dataset is shown in Fig. 4e of the main text; corresponding analyses excluding POFE1s and POFE2s are shown in Figs. S12e and S11e, respectively. See Sect. 2.1 for details on meteorological parameters elevation indexing.*

| Variable | Whole data | | Excluding POFE1 | | Excluding POFE2 | |
|---|---|---|---|---|---|---|
| | F1 (34.75%) | F2 (28.57%) | F1 (34.75%) | F2 (28.57%) | F1 (34.75%) | F2 (28.57%) |
| $V_{dw,O_3}$ | -0.19 | -0.44 | -0.19 | -0.44 | -0.19 | -0.44 |
| $u_*$ | 0.90 | -0.39 | 0.90 | -0.39 | 0.90 | -0.39 |
| $VPD_{8m}$ | 0.42 | 0.85 | 0.42 | 0.85 | 0.42 | 0.85 |
| $T_{8m}$ | 0.48 | 0.80 | 0.48 | 0.80 | 0.48 | 0.80 |
| $RH_{8m}$ | 0.12 | -0.35 | 0.12 | -0.35 | 0.12 | -0.35 |
| $WS_{8m}$ | 0.85 | -0.04 | 0.85 | -0.04 | 0.85 | -0.04 |
| $WS_{103m}$ | 0.89 | -0.41 | 0.89 | -0.41 | 0.89 | -0.41 |
| GSR | -0.07 | 0.53 | -0.07 | 0.53 | -0.07 | 0.53 |

***(f)*** *Factor loadings from PCA performed for daytime conditions under RH ≥ 40%. PCA visualization of full dataset is shown in Fig. 4f of the main text; corresponding analyses excluding POFE1s and POFE2s are shown in Figs. S12f and S11f, respectively. See Sect. 2.1 for details on meteorological parameters elevation indexing.*

| Variable | Whole data | | Excluding POFE1 | | Excluding POFE2 | |
|---|---|---|---|---|---|---|
| | F1 (45.37%) | F2 (27.29%) | F1 (45.37%) | F2 (27.29%) | F1 (44.85%) | F2 (27.90%) |
| $V_{dw,O_3}$ | -0.07 | 0.18 | -0.07 | 0.18 | -0.17 | 0.20 |
| $u_*$ | 0.66 | 0.74 | 0.66 | 0.74 | 0.64 | 0.75 |
| $VPD_{8m}$ | 0.87 | -0.41 | 0.87 | -0.41 | 0.88 | -0.40 |
| $T_{8m}$ | 0.78 | -0.44 | 0.78 | -0.44 | 0.78 | -0.44 |
| $RH_{8m}$ | -0.80 | 0.31 | -0.80 | 0.31 | -0.81 | 0.30 |
| $WS_{8m}$ | 0.78 | 0.34 | 0.78 | 0.34 | 0.77 | 0.36 |
| $WS_{103m}$ | 0.64 | 0.75 | 0.64 | 0.75 | 0.63 | 0.76 |
| GSR | 0.40 | -0.68 | 0.40 | -0.68 | 0.38 | -0.68 |

Figure 5 in the main text illustrates the relationship between $RH_{8m}$ and the deviation of various measured environmental variables from their overall means. Figure S14 provides an alternative visualization of the same data.

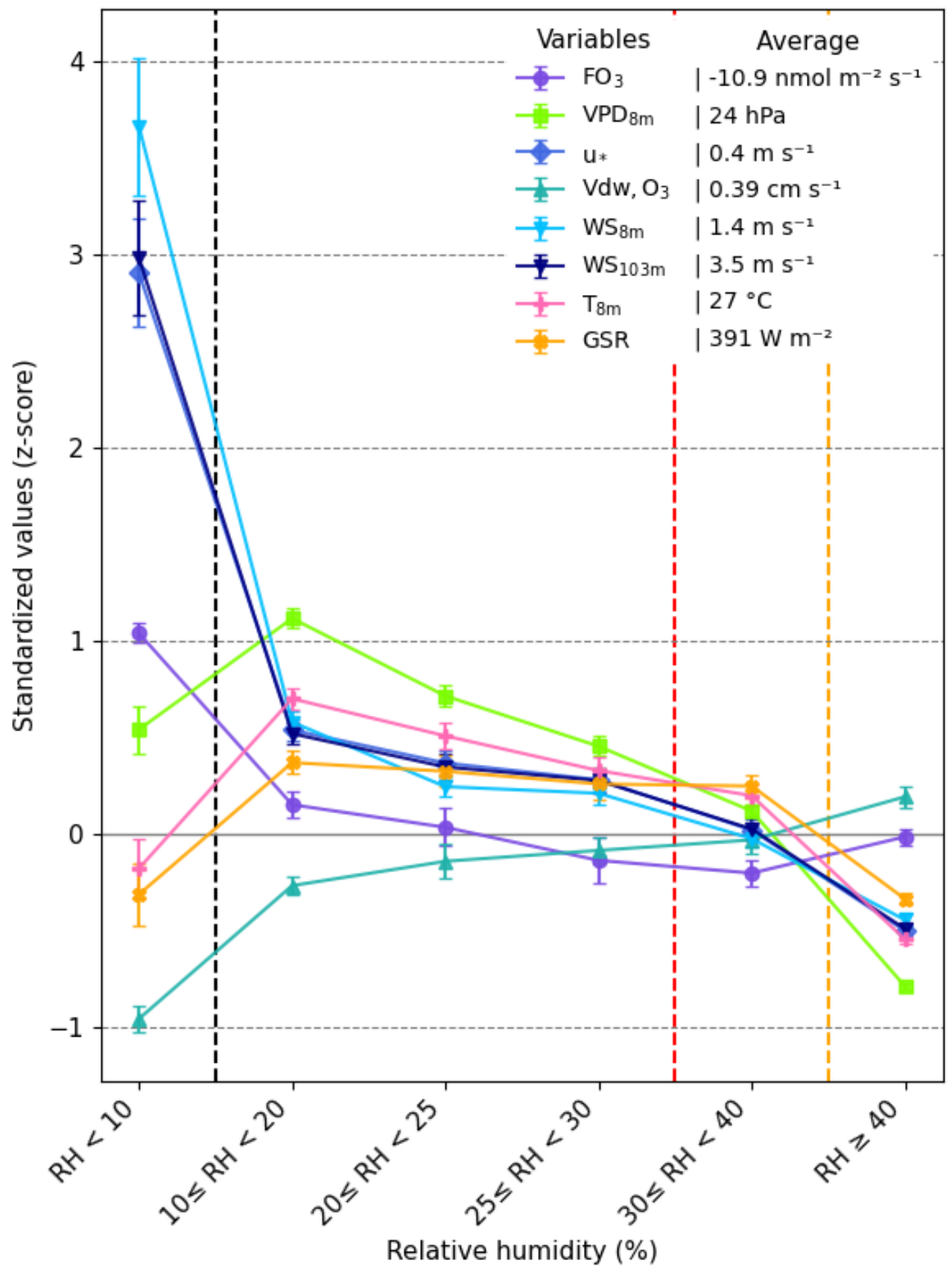


***Fig. S14.*** *Standardized (z-score) profiles of $O_3$ flux ($FO_3$), downward velocity of ozone ($Vdw,O_3$), wind speed (WS), temperature (T), vapor-pressure deficit (VPD), global solar radiation (GSR) and the friction velocity ($u_*$) across the bin-based relative humidity ($RH_{8m}$) ranges during the daytime. Horizontal bars indicate the mean z-score for each RH bin; black ticks indicate SEM. Dashed red line shows the $RH_{8m}$ threshold (< 30%) used to define POFE1, and the dashed orange line indicates an extra split within the left $RH_{8m}$ group (≥ 30%). Dashed black line shows $RH_{8m}$ < 10%. Z-scores were computed as $(x - \bar{x})/\sigma$, where $\bar{x}$ and $\sigma$ are the mean and standard deviation of the variable, respectively. The standardized value, expressed in units of $\sigma$, indicates how far each RH-bin mean of each variable deviates from the overall daytime mean, with positive and negative values showing deviations above and below the mean, respectively. This mean value is shown in the upper-right legend (Average). See Sect. 2.1 for details on meteorological parameters elevation indexing.*

Analyses presented in Fig. 5 in the main text indicate that $T_{8m}$ and GSR generally decreased with increasing $Vdw,O_3$ and RH bins. They were exceptionally low in the $RH_{8m} < 10\%$ bin, whereas $WS_{8m}$, $WS_{103m}$ and $u_*$ were exceptionally high in that bin. This pattern suggests that

elevated wind speeds may have contributed to reduced Vdw,$O_3$ under $RH_{8m}$ < 10% by enhancing surface evaporation and thereby reducing surface wetness.

**S4. Relevance of $NO_x$ and volatile organic compounds (VOCs) emissions to Vdw,$O_3$: PCA-based covariance**

PCA-based covariance analysis was performed to identify how $NO_x$ and VOC fluxes covary with Vdw,$O_3$, separately for daytime and nighttime periods, and the results are summarized in Fig. S15. In both periods, $NO_x$ showed stronger and more coherent covariation with Vdw,$O_3$ than any of the VOC groups. During daytime, the highest positive covariation coefficients with Vdw,$O_3$ were observed for NO (0.51) and $NO_2$ (0.52), while VOC coefficients were generally weak, ranging from slightly negative to modestly positive values.

During nighttime, the covariations of both $NO_x$ and the investigated VOCs were lower than during daytime. NO was the only species that showed substantial positive covariation with Vdw,$O_3$ (0.47) similar to its daytime value, and it remained substantially higher than all other nighttime covariations. The covariation of $NO_2$ was also significantly higher than that of any of the investigated VOCs. Note that the negative covariation of some VOCs, such as methyl ethyl ketone, acetone, propanal, C8 aromatics, and pentanones + pentanal, with Vdw,$O_3$ is unlikely to result from inhibition of $O_3$ deposition by emissions from elevated sources. This is supported by the fact that these compounds are predominantly surface-emitted, and their positive fluxes strongly dominate, even for those showing relatively stronger negative covariation at night.

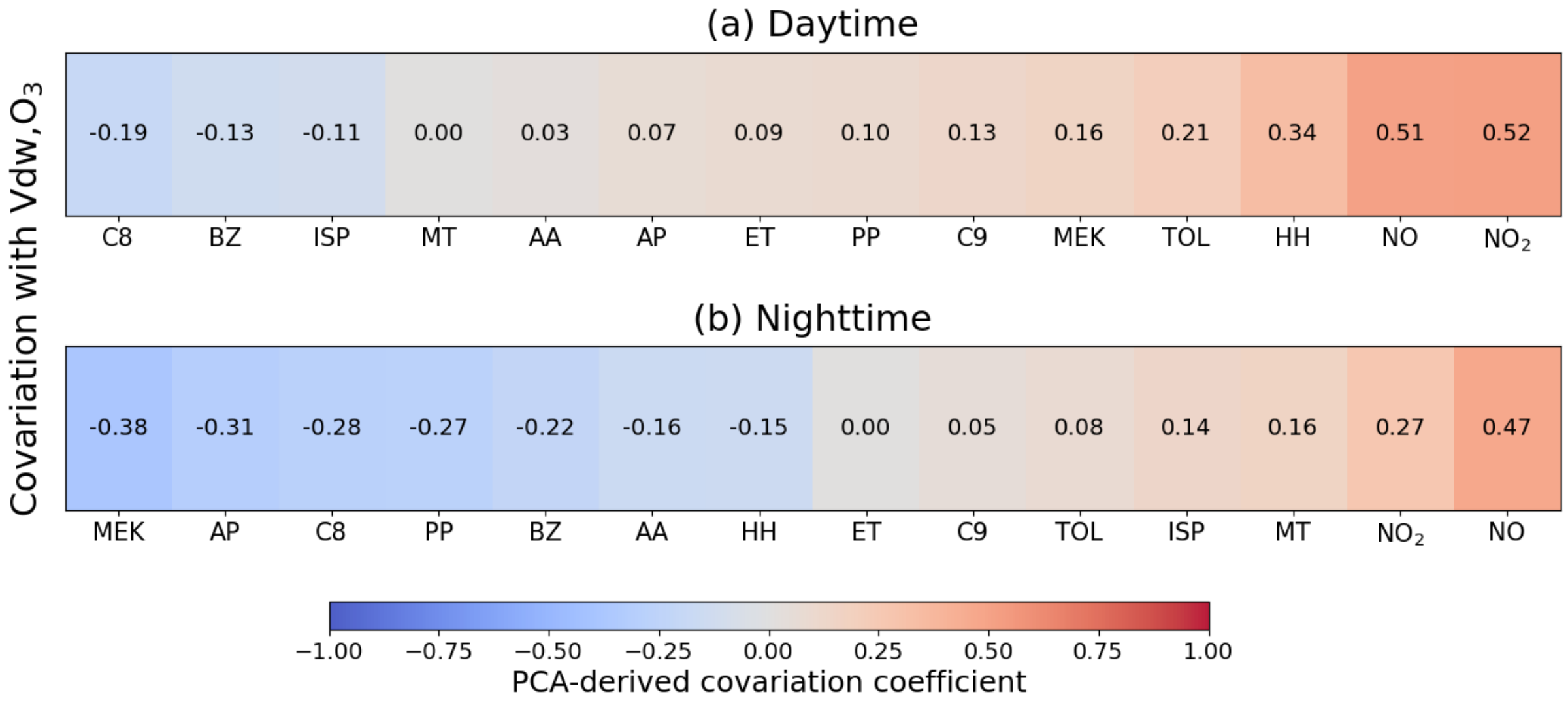


***Fig. S15.*** *PCA-derived covariation coefficients between downward ozone velocity (Vdw,$O_3$) and pollutant fluxes. Coefficients were derived from the factor loadings in Table S6 as the sum of the products of the loadings of Vdw,$O_3$ and each pollutant variable on the first two principal components (F1 and F2). The color scale ranges from −1 to 1, as indicated by the color bar (blue = negative, red = positive). Results are shown for (a) daytime and (b) nighttime. Abbreviations: AA (acetaldehyde), ET (ethanol), AP (acetone + propanal), MEK (methyl ethyl ketone + butanals), C8 (C8 aromatics), ISP (isoprene), BZ (benzene), PP (pentanones + pentanal), TOL (toluene), HH (hexanones + hexanal), C9 (C9 aromatics), and MT (monoterpenes).*

***Table S6.***

***(a)*** *Factor loadings from the PCA performed for daytime $NO_x$ and VOC fluxes with Vdw,$O_3$ are shown. PCA-derived covariation coefficients were calculated as the sum of the products of the loadings of Vdw,$O_3$ and each pollutant variable on the first two principal components (F1 and F2). The corresponding color scale spans −1 to 1. Abbreviations: AA (acetaldehyde), ET (ethanol), AP (acetone + propanal), MEK (methyl ethyl ketone + butanals), C8 (C8 aromatics), ISP (isoprene), BZ (benzene), PP (pentanones + pentanal), TOL (toluene), HH (hexanones + hexanal), C9 (C9 aromatics), and MT (monoterpenes).*

| **Variable** | **F1** (42.5%) | **F2** (13.4%) | **PCA-derived covariation coefficients with Vdw,$O_3$** |
|---|---|---|---|
| NO | 0.098 | 0.742 | 0.51 |
| $NO_2$ | 0.497 | 0.665 | 0.52 |
| Vdw,$O_3$ | 0.151 | 0.668 | |
| AA | 0.785 | -0.127 | 0.03 |
| ET | 0.658 | -0.012 | 0.09 |
| AP | 0.861 | -0.088 | 0.07 |
| MEK | 0.666 | 0.086 | 0.16 |
| C8 | 0.335 | -0.354 | -0.19 |
| ISP | 0.771 | -0.342 | -0.11 |
| BZ | 0.470 | -0.304 | -0.13 |
| PP | 0.901 | -0.055 | 0.10 |
| TOL | 0.557 | 0.194 | 0.21 |
| HH | 0.883 | -0.008 | 0.13 |
| C9 | 0.640 | 0.371 | 0.34 |
| MT | 0.787 | -0.180 | 0.00 |

***(b)*** *Factor loadings from the PCA performed for nighttime $NO_x$ and VOC fluxes with $Vdw,O_3$ are shown. PCA-derived covariation coefficients were calculated as the sum of the products of the loadings of $Vdw,O_3$ and each pollutant variable on the first two principal components (F1 and F2). The corresponding color scale spans −1 to 1. Abbreviations: AA (acetaldehyde), ET (ethanol), AP (acetone + propanal), MEK (methyl ethyl ketone + butanals), C8 (C8 aromatics), ISP (isoprene), BZ (benzene), PP (pentanones + pentanal), TOL (toluene), HH (hexanones + hexanal), C9 (C9 aromatics), and MT (monoterpenes).*

| **Variable** | **F1** (49.2%) | **F2** (14.4%) | **PCA-derived covariation coefficients with $Vdw,O_3$** |
|---|---|---|---|
| NO | -0.069 | 0.693 | 0.47 |
| $NO_2$ | 0.627 | 0.513 | 0.27 |
| $Vdw,O_3$ | -0.110 | 0.665 | |
| AA | 0.934 | -0.086 | -0.16 |
| ET | 0.884 | 0.140 | 0.00 |
| AP | 0.739 | -0.342 | -0.31 |
| MEK | 0.725 | -0.451 | -0.38 |
| C8 | 0.281 | -0.381 | -0.28 |
| ISP | 0.592 | 0.313 | 0.14 |
| BZ | 0.523 | -0.248 | -0.22 |
| PP | 0.840 | -0.263 | -0.27 |
| TOL | 0.782 | 0.249 | 0.08 |
| HH | 0.911 | -0.074 | -0.15 |
| C9 | 0.912 | 0.222 | 0.05 |
| MT | 0.751 | 0.371 | 0.16 |

## S5. Horizontal $O_3$ advection sensitivity analyses

To assess whether horizontal $O_3$ advection could influence the interpretation of $O_3$ fluxes measured at the EC height of 103 m, we performed two complementary sensitivity analyses. First, we estimated the horizontal $O_3$ gradient required for the vertically integrated horizontal-advection term between the surface and 103 m to become comparable in magnitude to observed $O_3$ flux magnitudes (Sect. S5.1). Second, we examined whether height-dependent differences in horizontal advection could modify vertical $O_3$ concentration differences between selected heights (Sect. S5.2).

### S5.1 Vertically integrated horizontal-advection budget analysis

We estimated the magnitude of the vertically integrated horizontal-advection term as

$$|F_{adv}| \approx 0.001 \cdot n_{air} \cdot G_{O_3} \cdot \int_0^{103} U(z)dz$$

where $F_{adv}$ is the estimated magnitude of the vertically integrated horizontal-advection contribution (nmol $m^{-2}$ $s^{-1}$), $n_{air}$ is the molar density of air (mol $m^{-3}$), $GO_3$ is the assumed magnitude of the horizontal $O_3$ concentration gradient (ppb $km^{-1}$), and U(z) is the observed horizontal wind speed at height z (m $s^{-1}$). The vertically integrated wind speed, $\int_0^{103} U(z)dz$ has units of $m^2$ $s^{-1}$. The factor 0.001 accounts for the unit conversion required to express $F_{adv}$ in nmol $m^{-2}$ $s^{-1}$. Wind-speed measurements at 8, 15, 32, 47, 65, 80, and 103 m were used to estimate the vertical wind-speed profile between the surface and 103 m. Wind speed at the surface was assumed to be zero. The resulting vertical profile was integrated over the full 0–103 m height range using the trapezoidal method.

For this magnitude-based sensitivity calculation, maximum alignment between the horizontal wind and the horizontal $O_3$ concentration-gradient was assumed. Accordingly, the observed wind direction was not explicitly used in the calculation, and only the wind-speed magnitude was considered.

Because the horizontal $O_3$ gradient was not measured, we instead calculated the gradient required for the vertically integrated horizontal-advection term to reach selected reference flux

magnitudes. Accordingly, we calculated the horizontal $O_3$ gradient required for the magnitude of the vertically integrated horizontal-advection term to equal a specified reference vertical $O_3$ flux magnitude as

$$G_{crit} = \frac{F_{ref}}{0.001 \cdot n_{air} \cdot \int_0^{103} U(z)dz}$$

where $G_{crit}$ is the critical horizontal $O_3$ gradient (ppb km$^{-1}$), and $F_{ref}$ is the selected reference vertical $O_3$ flux magnitude. We used reference flux magnitudes based on $O_3$ flux measurements at the eddy covariance (EC) measurement height: 5.24 and 11.66 nmol m$^{-2}$ s$^{-1}$. The former represents the mean positive $O_3$ flux during POFE2 periods, i.e., positive ozone-flux events (POFEs) associated with downward NO flux (FNO < 0), whereas the latter represents the magnitude of the mean daytime downward $O_3$ flux.

These reference values are based on $O_3$ fluxes measured at 103 m and are not estimates of the surface $O_3$ flux or of the difference between the surface and the flux at 103 m. The actual horizontal $O_3$ gradient was also not measured. Accordingly, the calculation provides a magnitude-based sensitivity estimate and does not determine the magnitude or direction of the actual horizontal-advection contribution. A smaller degree of alignment between the wind and the $O_3$ gradient would correspond to a larger $O_3$ gradient for the same advective contribution.

For all daytime observations, the median vertically integrated wind speed was 227.8 m$^2$ s$^{-1}$, corresponding to critical $O_3$ gradients of approximately 0.56 and 1.25 ppb km$^{-1}$ for reference flux magnitudes of 5.24 and 11.66 nmol m$^{-2}$ s$^{-1}$, respectively. For POFE2 periods, the corresponding critical gradients were approximately 1.01 and 2.24 ppb km$^{-1}$. When only daytime POFE2s were considered, the values were nearly unchanged, 1.02 and 2.28 ppb km$^{-1}$, respectively. Thus, under the assumptions of this sensitivity calculation, horizontal $O_3$ gradients of approximately 0.6–2.3 ppb km$^{-1}$ could produce a vertically integrated horizontal-advection contribution of the same order of magnitude as the selected reference $O_3$ flux magnitudes. Because the actual horizontal $O_3$ gradient was not measured, evaluating the relevance of these sensitivity estimates requires assessing whether horizontal $O_3$ gradients of this magnitude are realistic.

The critical-gradient values obtained here, approximately 0.6–2.3 ppb km$^{-1}$, are comparable in magnitude to reported horizontal $O_3$ gradients and plume-scale $O_3$ concentration differences in urban plumes and plume-influenced environments. For example, aircraft measurements downwind of Milan showed afternoon $O_3$ gradients of approximately 0.6–1.0 ppb

km$^{-1}$. In a Berlin urban plume, $O_3$ concentrations were approximately 15 ppb higher inside than outside a plume about 40 km wide.[5] Assuming that this difference in $O_3$ concentration between inside and outside the plume developed over approximately half of the plume width (~20 km), it would correspond to an average horizontal $O_3$ gradient of approximately 0.75 ppb km$^{-1}$, which falls within the critical-gradient range estimated here.

Although the Berlin value represents a plume-scale average estimate rather than a directly reported gradient, this estimate, together with the Milan observations, indicates that horizontal $O_3$ gradients within the range estimated in our critical-gradient analysis can occur in urban and plume-influenced environments.

These comparisons therefore indicate that such horizontal $O_3$ gradients could produce a vertically integrated horizontal-advection contribution of the same order of magnitude as the observed positive $O_3$ fluxes during POFE2 periods. While the combined analyses presented in Sect. S2.2 provide consistent support for our interpretation that higher-above-measurement NO concentrations (HAMNC), likely associated with elevated NO emissions, contributed to the observed $O_3$-fluxe behavior, our measurements do not allow us to determine the extent to which horizontal advection or other processes also contribute to the vertically integrated $O_3$ budget during these periods.

This limitation also prevents us from attributing any possible difference between the $O_3$ flux measured at 103 m and the actual surface flux to a specific process. NO–$O_3$ chemistry between the surface and the EC measurement height could contribute to a vertical difference between the $O_3$ flux at the surface and that measured at 103 m. Measurements at multiple heights would be required to directly evaluate such vertical flux divergence. Because our order-of-magnitude analysis indicates that horizontal advection could also contribute appreciably to the vertically integrated $O_3$ budget, a possible difference between the surface $O_3$ flux and the flux measured at 103 m cannot be assumed to be negligible. Because neither the surface $O_3$ flux nor the horizontal $O_3$ gradient was measured, the relative contributions of chemical flux divergence and horizontal advection cannot be quantified from our observations.

It is important to note that, while the order-of-magnitude analysis indicates that horizontal advection could be sufficiently large to contribute to positive $O_3$ fluxes at 103 m, our interpretation of the POFE2s and related reduction in observed downward $O_3$ velocity ($V_{dw,O_3}$) remains based on the combined evidence presented in Sect. S2.2. This evidence supports a contribution of

HAMNC, likely associated with NO emissions from elevated sources, to the occurrence of POFE2s and to reduced $V_{dw,O_3}$, and emphasizes the need to consider elevated-source NO emissions when interpreting urban $O_3$ flux measurements.

## S5.2 Differential horizontal-advection sensitivity analysis

Given that the vertically integrated budget analysis indicated that horizontal advection could contribute appreciably to the $O_3$ budget, we further examined whether differences in horizontal advection between heights could modify the vertical $O_3$ concentration profile and, in turn, the $O_3$ flux measured at 103 m. Such differential horizontal advection could represent an additional factor affecting the measured $O_3$ flux, potentially reinforcing or counteracting the effects associated with HAMNC on downward $O_3$ fluxes and the occurrence of positive fluxes at 103 m, while also contributing to a difference between the surface $O_3$ flux and the flux measured at the EC height.

To examine this possibility, horizontal wind vectors were calculated for each 30-min period from the wind-speed and wind-direction measurements at the selected heights. Because the magnitude and direction of the horizontal $O_3$ gradient were not measured, the calculation was formulated as a sensitivity analysis rather than as a site-specific estimate. For each height pair, all nine combinations of horizontal $O_3$ gradient magnitudes of 0.5, 1.0, and 2.0 ppb $km^{-1}$ at the two heights were considered. The horizontal $O_3$ gradient was assumed to have the same direction at both heights, whereas its magnitude could differ.

For each 30-min period and each combination of horizontal $O_3$-gradient magnitudes at the two heights, we calculated the potential magnitude of the differential horizontal-advective tendency between the two heights as:

$$|\Delta A_h| = 3.6\ \|G_{h2}V_{h2} - G_{h1}V_{h1}\|$$

Here, $|\Delta Ah|$ is the magnitude of the difference between the horizontal-advective tendencies at the two heights, i.e., the difference in the rates of $O_3$ concentration change induced by horizontal advection, expressed in ppb $h^{-1}$. G is the assumed horizontal $O_3$ gradient magnitude, and V is the horizontal wind vector calculated from the measured wind speed and wind direction. The factor 3.6 converts m $s^{-1}$ × ppb $km^{-1}$ to ppb $h^{-1}$. Because the actual horizontal $O_3$-gradient direction was

not measured, the calculation does not necessarily represent the actual differential horizontal-advective tendency between the two heights in the rate of $O_3$ concentration change induced by horizontal advection. Rather, it provides an estimate of the potential magnitude of this differential tendency under the prescribed gradient scenarios. Based on this differential tendency, the corresponding potential change in the $O_3$ concentration difference over the 30-min averaging period was estimated as:

$$\Delta O_{3,30min}^{pot} = |\Delta A_h| \times 0.5\ h$$

Here, $\Delta O_{3,30min}^{pot}$ is the potential 30-min change in the $O_3$ concentration difference estimated from the sensitivity analysis, with the superscript “pot” indicating that this is not an observed $O_3$ concentration change.

We selected horizontal $O_3$ gradient magnitudes of 0.5, 1.0, and 2.0 ppb $km^{-1}$ for the differential-advection sensitivity analysis. These values span the critical-gradient range estimated from the vertically integrated advection calculation, whose relevance to urban and plume-influenced conditions was discussed above.[4,5]

We first examined the 8–103 m height pair, which spans the largest vertical separation between the available measurement heights below and at the EC measurement level (Table S7a). For this comparison, the median potential 30-min $O_3$ changes across the nine assumed horizontal-gradient combinations ranged from 1.80 to 10.20 ppb for all daytime observations, from 0.92 to 4.98 ppb for all POFE2 periods, and from 0.68 to 4.35 ppb for daytime POFE2 periods (Table S7a). Therefore, the differential-advection sensitivity estimates during POFE2 periods were generally smaller than those for all daytime conditions.

The differential-advection sensitivity analysis was also performed for additional height pairs (Tables S7a–b). Across the tested height pairs and horizontal $O_3$-gradient scenarios, differential horizontal advection could potentially modify vertical $O_3$ concentration differences by several ppb over 30 min. Because the direction of the horizontal $O_3$ gradient was not measured, however, the analysis does not indicate whether these changes would increase or decrease the vertical $O_3$ concentration difference. Differential-advection estimates during daytime POFE2 periods were generally smaller than, or at least not larger than, those for the full daytime dataset. Thus, the observed wind fields during POFE2 periods do not indicate enhanced differential horizontal advection relative to the broader daytime dataset.

***Table S7.***

***(a)** Height-pair differential-advection sensitivity results for the magnitude of the differential horizontal-advective tendency ($|\Delta A_h|$). For each height pair, the reported ranges for Q1, median, and Q3, corresponding to the 25th, 50th, and 75th percentiles, respectively, span the minimum and maximum values obtained across the nine combinations of assumed horizontal $O_3$ gradient magnitudes. $|\Delta A_h|$ is expressed in ppb $h^{-1}$.*

| Subset | Pair group | Height pair | n | $\|\Delta A_h\|$ (ppb $h^{-1}$) | | |
|---|---|---|---|---|---|---|
| | | | | Q1 range | Median range | Q3 range |
| All daytime | 103 m-referenced | 8-103 m | 1637 | 1.88-13.36 | 3.60-20.40 | 7.58-31.92 |
| | | 15-103 m | 1637 | 3.78-15.10 | 5.55-22.22 | 8.39-33.57 |
| | | 32-103 m | 1636 | 2.14-12.60 | 3.47-19.07 | 5.75-29.51 |
| | | 47-103 m | 1637 | 1.07-11.87 | 1.97-18.02 | 3.61-27.79 |
| | | 65-103 m | 1635 | 1.48-11.77 | 2.36-17.67 | 3.71-27.08 |
| | | 80-103 m | 1637 | 1.35-11.63 | 2.03-17.26 | 3.10-26.37 |
| | Adjacent below 103 m | 8-15 m | 1813 | 2.27-9.07 | 3.27-13.08 | 4.53-18.11 |
| | | 15-32 m | 1811 | 1.79-9.90 | 2.66-13.30 | 3.99-17.12 |
| | | 32-47 m | 1810 | 1.04-9.67 | 1.57-13.08 | 2.40-17.19 |
| | | 47-65 m | 1808 | 0.64-10.35 | 1.03-14.69 | 1.61-20.43 |
| | | 65-80 m | 1808 | 0.28-10.54 | 0.53-15.15 | 0.99-22.17 |
| | Extended above 103 m | 47-140 m | 1810 | 1.05-12.24 | 2.11-19.32 | 4.42-32.25 |
| | | 80-120 m | 1776 | 0.75-11.86 | 1.25-18.12 | 2.48-28.46 |
| | | 80-140 m | 1810 | 1.01-11.94 | 1.69-18.58 | 3.25-30.44 |
| | | 103-120 m | 1636 | 0.81-11.52 | 1.29-17.55 | 1.93-27.43 |
| | | 103-140 m | 1636 | 0.68-11.47 | 1.24-18.04 | 1.98-29.37 |
| All POFE2 | 103 m-referenced | 8-103 m | 28 | 1.13-7.46 | 1.83-9.96 | 3.45-15.18 |
| | | 15-103 m | 28 | 2.31-9.23 | 3.20-12.80 | 4.65-18.60 |
| | | 32-103 m | 28 | 1.26-7.01 | 1.83-9.65 | 2.78-13.44 |
| | | 47-103 m | 28 | 0.56-6.47 | 1.13-8.92 | 2.05-12.68 |
| | | 65-103 m | 28 | 0.91-6.74 | 1.40-8.96 | 2.22-12.61 |
| | | 80-103 m | 28 | 0.79-6.15 | 1.12-8.95 | 1.62-12.53 |
| | Adjacent below 103 m | 8-15 m | 29 | 1.25-4.99 | 1.86-7.43 | 2.44-9.76 |
| | | 15-32 m | 29 | 1.39-5.95 | 1.88-7.51 | 2.16-9.91 |
| | | 32-47 m | 29 | 0.70-5.46 | 1.05-7.74 | 1.33-10.14 |
| | | 47-65 m | 29 | 0.31-5.77 | 0.62-8.31 | 0.83-10.23 |
| | | 65-80 m | 29 | 0.18-6.62 | 0.31-9.20 | 0.58-10.91 |
| | Extended above 103 m | 47-140 m | 29 | 0.56-5.68 | 1.13-9.28 | 2.03-14.13 |
| | | 80-120 m | 28 | 0.47-5.91 | 0.86-8.57 | 1.35-12.57 |
| | | 80-140 m | 29 | 0.80-6.50 | 1.27-9.23 | 1.88-13.37 |
| | | 103-120 m | 28 | 0.36-6.12 | 0.56-8.75 | 0.96-12.56 |
| | | 103-140 m | 28 | 0.37-6.39 | 0.55-8.76 | 1.10-12.41 |
| Daytime POFE2 | 103 m-referenced | 8-103 m | 16 | 0.96-7.26 | 1.35-8.69 | 1.86-12.35 |
| | | 15-103 m | 16 | 2.60-10.41 | 3.08-12.31 | 3.62-14.48 |
| | | 32-103 m | 16 | 1.17-6.89 | 1.50-8.18 | 1.95-11.79 |
| | | 47-103 m | 16 | 0.39-6.47 | 0.84-7.61 | 1.16-10.80 |
| | | 65-103 m | 16 | 0.84-6.45 | 1.08-7.85 | 1.66-10.99 |
| | | 80-103 m | 16 | 0.75-6.65 | 1.05-8.01 | 1.39-10.76 |
| | Adjacent below 103 m | 8-15 m | 16 | 1.80-7.19 | 2.22-8.86 | 2.62-10.48 |
| | | 15-32 m | 16 | 1.63-6.51 | 1.88-7.90 | 2.07-9.93 |
| | | 32-47 m | 16 | 0.70-6.13 | 0.92-7.71 | 1.17-9.91 |
| | | 47-65 m | 16 | 0.29-6.14 | 0.44-7.53 | 0.69-9.81 |
| | | 65-80 m | 16 | 0.11-6.46 | 0.22-7.73 | 0.30-9.63 |
| | Extended above 103 m | 47-140 m | 16 | 0.55-6.11 | 0.86-7.98 | 1.29-10.67 |
| | | 80-120 m | 16 | 0.47-6.69 | 0.66-7.80 | 0.96-10.98 |
| | | 80-140 m | 16 | 0.77-6.74 | 1.08-8.17 | 1.39-10.74 |
| | | 103-120 m | 16 | 0.29-6.12 | 0.54-7.56 | 0.85-10.71 |
| | | 103-140 m | 16 | 0.37-6.39 | 0.54-7.81 | 0.76-10.55 |

**Note**. Sample size varies among height pairs according to the availability of wind data at the respective heights. One POFE2 period was excluded from comparisons involving 103 m because the required wind data at 103 m were unavailable.

***(b)*** *Height–pair differential–advection sensitivity results for the potential 30–min change in the $O_3$ concentration difference ($\Delta O_3$,30min$^{pot}$). For each height pair, the reported ranges for Q1, median, and Q3, corresponding to the 25th, 50th, and 75th percentiles, respectively, span the minimum and maximum values obtained across the nine combinations of assumed horizontal $O_3$ gradient magnitudes. $\Delta O_3$,30min$^{pot}$ is expressed in ppb.*

| Subset | Pair group | Height pair | n | $\Delta O_3$,30min$^{pot}$ (ppb) | | |
|---|---|---|---|---|---|---|
| | | | | Q1 range | Median range | Q3 range |
| All daytime | 103 m-referenced | 8-103 m | 1637 | 0.94-6.68 | 1.80-10.20 | 3.79-15.96 |
| | | 15-103 m | 1637 | 1.89-7.55 | 2.78-11.11 | 4.20-16.79 |
| | | 32-103 m | 1636 | 1.07-6.30 | 1.74-9.54 | 2.88-14.75 |
| | | 47-103 m | 1637 | 0.54-5.93 | 0.98-9.01 | 1.80-13.89 |
| | | 65-103 m | 1635 | 0.74-5.89 | 1.18-8.83 | 1.85-13.54 |
| | | 80-103 m | 1637 | 0.67-5.82 | 1.02-8.63 | 1.55-13.19 |
| | Adjacent below 103 m | 8-15 m | 1813 | 1.13-4.53 | 1.63-6.54 | 2.26-9.05 |
| | | 15-32 m | 1811 | 0.90-4.95 | 1.33-6.65 | 2.00-8.56 |
| | | 32-47 m | 1810 | 0.52-4.83 | 0.79-6.54 | 1.20-8.59 |
| | | 47-65 m | 1808 | 0.32-5.17 | 0.52-7.34 | 0.81-10.22 |
| | | 65-80 m | 1808 | 0.14-5.27 | 0.26-7.57 | 0.49-11.09 |
| | Extended above 103 m | 47-140 m | 1810 | 0.53-6.12 | 1.06-9.66 | 2.21-16.13 |
| | | 80-120 m | 1776 | 0.37-5.93 | 0.62-9.06 | 1.24-14.23 |
| | | 80-140 m | 1810 | 0.50-5.97 | 0.85-9.29 | 1.62-15.22 |
| | | 103-120 m | 1636 | 0.41-5.76 | 0.64-8.77 | 0.97-13.72 |
| | | 103-140 m | 1636 | 0.34-5.74 | 0.62-9.02 | 0.99-14.68 |
| All POFE2 | 103 m-referenced | 8-103 m | 28 | 0.57-3.73 | 0.92-4.98 | 1.73-7.59 |
| | | 15-103 m | 28 | 1.15-4.61 | 1.60-6.40 | 2.33-9.30 |
| | | 32-103 m | 28 | 0.63-3.50 | 0.92-4.82 | 1.39-6.72 |
| | | 47-103 m | 28 | 0.28-3.24 | 0.57-4.46 | 1.03-6.34 |
| | | 65-103 m | 28 | 0.45-3.37 | 0.70-4.48 | 1.11-6.31 |
| | | 80-103 m | 28 | 0.39-3.08 | 0.56-4.47 | 0.81-6.26 |
| | Adjacent below 103 m | 8-15 m | 29 | 0.62-2.49 | 0.93-3.72 | 1.22-4.88 |
| | | 15-32 m | 29 | 0.70-2.98 | 0.94-3.76 | 1.08-4.96 |
| | | 32-47 m | 29 | 0.35-2.73 | 0.52-3.87 | 0.66-5.07 |
| | | 47-65 m | 29 | 0.16-2.88 | 0.31-4.15 | 0.42-5.11 |
| | | 65-80 m | 29 | 0.09-3.31 | 0.15-4.60 | 0.29-5.46 |
| | Extended above 103 m | 47-140 m | 29 | 0.28-2.84 | 0.57-4.64 | 1.01-7.06 |
| | | 80-120 m | 28 | 0.24-2.95 | 0.43-4.28 | 0.67-6.29 |
| | | 80-140 m | 29 | 0.40-3.25 | 0.64-4.61 | 0.94-6.69 |
| | | 103-120 m | 28 | 0.18-3.06 | 0.28-4.38 | 0.48-6.28 |
| | | 103-140 m | 28 | 0.18-3.20 | 0.28-4.38 | 0.55-6.21 |
| Daytime POFE2 | 103 m-referenced | 8-103 m | 16 | 0.48-3.63 | 0.68-4.35 | 0.93-6.18 |
| | | 15-103 m | 16 | 1.30-5.21 | 1.54-6.15 | 1.81-7.24 |
| | | 32-103 m | 16 | 0.59-3.44 | 0.75-4.09 | 0.98-5.89 |
| | | 47-103 m | 16 | 0.19-3.24 | 0.42-3.81 | 0.58-5.40 |
| | | 65-103 m | 16 | 0.42-3.22 | 0.54-3.93 | 0.83-5.49 |
| | | 80-103 m | 16 | 0.38-3.32 | 0.53-4.00 | 0.70-5.38 |
| | Adjacent below 103 m | 8-15 m | 16 | 0.90-3.60 | 1.11-4.43 | 1.31-5.24 |
| | | 15-32 m | 16 | 0.81-3.25 | 0.94-3.95 | 1.04-4.96 |
| | | 32-47 m | 16 | 0.35-3.07 | 0.46-3.85 | 0.59-4.95 |
| | | 47-65 m | 16 | 0.14-3.07 | 0.22-3.76 | 0.34-4.91 |
| | | 65-80 m | 16 | 0.05-3.23 | 0.11-3.87 | 0.15-4.82 |
| | Extended above 103 m | 47-140 m | 16 | 0.28-3.06 | 0.43-3.99 | 0.64-5.34 |
| | | 80-120 m | 16 | 0.24-3.35 | 0.33-3.90 | 0.48-5.49 |
| | | 80-140 m | 16 | 0.38-3.37 | 0.54-4.08 | 0.70-5.37 |
| | | 103-120 m | 16 | 0.14-3.06 | 0.27-3.78 | 0.43-5.35 |
| | | 103-140 m | 16 | 0.18-3.20 | 0.27-3.90 | 0.38-5.28 |

Note. Sample size varies among height pairs according to the availability of wind data at the respective heights. One POFE2 period was excluded from comparisons involving 103 m because the required wind data at 103 m were unavailable.

### S6. $O_3$ random-flux uncertainty assessment and robustness analyses

Because positive $O_3$ fluxes are atypical in the context of surface-layer $O_3$ dry deposition, we evaluated whether the positive $O_3$ fluxes defining POFEs were distinguishable from zero relative to their corresponding random-flux uncertainties. We also repeated key analyses after applying a conservative uncertainty-screening criterion, $|FO_3| > 2\sigma_F$, to determine whether the main conclusions were affected by excluding $O_3$ flux observations that did not satisfy this criterion. The random-flux uncertainty was calculated from the high-frequency turbulent fluctuations in vertical wind velocity and $O_3$ concentration using the random-shuffle method of Billesbach.[6] In this method, one of these high-frequency time series is randomly reordered before recalculating the covariance, thereby disrupting the coherent turbulent covariance and allowing the random covariance contribution to be estimated. For each 30-min period, we compared the magnitude of the measured $O_3$ flux, $|FO_3|$, with the corresponding $O_3$-specific random-flux uncertainty estimate, $\sigma_F$. Here, $\sigma_F$ represents one standard deviation ($1\sigma$) of the random-flux uncertainty estimated by the Billesbach[6] method. This estimate reflects the random-error component captured by the random-shuffle analysis and does not include other sources of uncertainty in the $O_3$ flux measurement. We performed this comparison for the full quality-controlled dataset (flux quality index, $Q_{class} \leq 6$) to provide a reference for the magnitude of the random-flux uncertainty relative to the measured $O_3$ fluxes, and separately for all POFEs and for the POFE1 (POFEs with RH < 30%) and POFE2 subsets.

We examined both $|FO_3| > \sigma_F$, and the more conservative criterion $|FO_3| > 2\sigma_F$. For the POFE subsets, these comparisons were used to assess whether the positive $O_3$ fluxes were distinguishable from zero relative to their corresponding $O_3$-specific random-flux uncertainty.

***Table S8***

***(a)** Assessment of $O_3$ fluxes during POFEs using random-flux uncertainty estimates. The analysis was performed for all POFEs and separately for the POFE1 and POFE2 subsets. The full quality-controlled dataset is also included as a reference (All data) for comparison with the POFE subsets. Only periods satisfying the quality criterion $Q_{class} \leq 6$ were included in any of the analyzed groups. $FO_3$ denotes the measured $O_3$ flux, and $\sigma_F$ denotes the corresponding $O_3$-specific random-flux uncertainty estimate for each 30-min period, calculated using the random-shuffle method of Billesbach.[6] Percentages in parentheses indicate the fraction of periods satisfying each uncertainty threshold.*

| Criterion | All data | All POFEs | POFE1 | POFE2 |
|---|---|---|---|---|
| Valid data ($Q_{class} \leq 6$) | 1306 | 123 | 46 | 29 |
| POFE ($FO_3 > 0$) | 123 | 123 | 46 | 29 |
| $\|FO_3\| > \sigma_F$ | 1296 (99.2%) | 117 (95.1%) | 42 (91.3%) | 28 (96.6%) |
| $\|FO_3\| > 2\sigma_F$ | 1281 (98.1%) | 109 (88.6%) | 40 (87.0%) | 26 (89.7%) |
| Median $\|FO_3\|$ (nmol $m^{-2}$ $s^{-1}$) | 8.84 | 1.67 | 1.98 | 1.28 |
| Median $\sigma_F$ (nmol $m^{-2}$ $s^{-1}$) | 0.27 | 0.14 | 0.11 | 0.19 |
| Median of $\|FO_3\| / \sigma_F$ | 32.0 | 12.6 | 19.1 | 11.9 |

***(b)** Assessment of $O_3$ fluxes during daytime POFEs using random-flux uncertainty estimates. The analysis was performed for all daytime POFEs and separately for the corresponding POFE1 and POFE2 subsets. The full quality-controlled dataset is also included as a reference (All data) for comparison with the POFE subsets. Only periods satisfying the quality criterion $Q_{class} \leq 6$ were included in any of the analyzed groups. $FO_3$ denotes the measured $O_3$ flux, and $\sigma_F$ denotes the corresponding $O_3$-specific random-flux uncertainty estimate for each 30-min period, calculated using the random-shuffle method of Billesbach.[6] Percentages in parentheses indicate the fraction of periods satisfying each uncertainty threshold.*

| Criterion | All data | All POFEs | POFE1 | POFE2 |
|---|---|---|---|---|
| Valid data ($Q_{class} \leq 6$) | 1119 | 93 | 45 | 16 |
| POFE ($FO_3 > 0$) | 93 | 93 | 45 | 16 |
| $\|FO_3\| > \sigma_F$ | 1111 (99.3%) | 88 (94.6%) | 41 (91.1%) | 15 (93.8%) |
| $\|FO_3\| > 2\sigma_F$ | 1099 (98.2%) | 83 (89.2%) | 39 (86.7%) | 14 (87.5%) |
| Median $\|FO_3\|$ (nmol $m^{-2}$ $s^{-1}$) | 9.69 | 1.91 | 2.04 | 1.33 |
| Median $\sigma_F$ (nmol $m^{-2}$ $s^{-1}$) | 0.28 | 0.12 | 0.12 | 0.19 |
| Median of $\|FO_3\| / \sigma_F$ | 32.9 | 17.2 | 20.3 | 13.1 |

***(c)** Assessment of $O_3$ fluxes during nighttime POFEs using random-flux uncertainty estimates. The analysis was performed for all nighttime POFEs and separately for the corresponding POFE1 and POFE2 subsets. The full quality-controlled dataset is also included as a reference (All data) for comparison with the POFE subsets. Only periods satisfying the quality criterion $Q_{class} \leq 6$ were included in any of the analyzed groups. $FO_3$ denotes the measured $O_3$ flux, and $\sigma_F$ denotes the corresponding $O_3$-specific random-flux uncertainty estimate for each 30-min period, calculated using the random-shuffle method of Billesbach.[6] Percentages in parentheses indicate the fraction of periods satisfying each uncertainty threshold.*

| Criterion | All data | All POFEs | POFE1 | POFE2 |
|---|---|---|---|---|
| Valid data ($Q_{class} \leq 6$) | 187 | 30 | 1 | 13 |
| POFE ($FO_3 > 0$) | 30 | 30 | 1 | 13 |
| $\|FO_3\| > \sigma_F$ | 185 (98.9%) | 29 (96.7%) | 1 (100.0%) | 13 (100.0%) |
| $\|FO_3\| > 2\sigma_F$ | 182 (97.3%) | 26 (86.7%) | 1 (100.0%) | 12 (92.3%) |
| Median $\|FO_3\|$ (nmol $m^{-2}$ $s^{-1}$) | 4.62 | 0.84 | 0.61 | 0.62 |
| Median $\sigma_F$ (nmol $m^{-2}$ $s^{-1}$) | 0.20 | 0.18 | 0.07 | 0.19 |
| Median of $\|FO_3\| / \sigma_F$ | 23.8 | 7.0 | 8.5 | 10.9 |

The results show that among the 123 POFEs, 117 cases (95.1%) exceeded their corresponding random-flux uncertainty estimate, and 109 cases (88.6%) also exceeded the more conservative $2\sigma_F$ criterion. For POFE2 specifically, 28 of 29 events (96.6%) exceeded $\sigma_F$, and 26 of 29 events (89.7%) exceeded $2\sigma_F$ (Table S8a).

The $|FO_3|/\sigma_F$ ratios further indicated that the positive $O_3$ fluxes were generally much larger than their corresponding random-flux uncertainty estimates. For all POFEs, the median $|FO_3|/\sigma_F$ ratio was 12.6, indicating that the measured positive $O_3$ flux typically exceeded its corresponding random-flux uncertainty estimate by more than an order of magnitude. For POFE2, the median $|FO_3|/\sigma_F$ ratio was 11.9, indicating that the positive $O_3$ flux during POFE2 periods was similarly much larger than the corresponding random-flux uncertainty estimate (Table S8a). The median $|FO_3|/\sigma_F$ ratio was lower for POFEs than for the full quality-controlled dataset (12.6 versus 32.0), which appears to mainly reflect the lower $O_3$ flux magnitudes during POFEs relative to the full dataset, rather than larger random-flux uncertainty estimates. Nevertheless, the median ratio remained greater than an order of magnitude for both all POFEs and POFE2 periods.

Overall, 95.1% of the positive $O_3$ fluxes used to define POFEs exceeded their corresponding $O_3$-specific random-flux uncertainty estimates, with the corresponding fraction reaching 96.6% for POFE2 periods. This indicates that the POFE and POFE2 classifications are robust with respect to the calculated random-flux uncertainty.

We further tested the robustness of several of the main conclusions of the manuscript by repeating the corresponding analyses after excluding $O_3$ flux observations that did not satisfy the more conservative $|FO_3|>2\sigma_F$ criterion. The results showed that the main conclusions were retained. Specifically: (i) after excluding POFE2-associated observations, the mean proportion of POFEs remained significantly higher for low relative humidity measured at 8 m (RH-low; $RH_{8m}<30\%$) than for $RH_{8m} \geq 30\%$; (ii) during daytime, the higher occurrence of POFEs under negative FNO than under non-negative FNO conditions remained statistically significant; (iii) the statistically significant positive relationship between Vdw,$O_3$ and FNO during both daytime and nighttime was retained; (iv) the logarithmic increase of Vdw,$O_3$ with increasing RH within the low-RH range (RH-low; $RH_{8m}<30\%$) and the hyperbolic increase within the high-RH range (RH-high; $RH_{8m}\geq70\%$) were retained, and (v) the logarithmic increase of Vdw,$O_3$ with FNO up to approximately 1.61 nmol $m^{-2}$ $s^{-1}$, including the FNO<0 range, and the hyperbolic increase between approximately 1.61 and 2.32 nmol $m^{-2}$ $s^{-1}$ were also retained. These sensitivity analyses therefore indicate that excluding $O_3$ flux observations that did not satisfy the $|FO_3|>2\sigma_F$ criterion did not change the main conclusions of the study.

**REFERENCES**


(1) Ryerson, T. B.; Trainer, M; Holloway, J. S.; Parrish, D. D.; Huey, L. G.; Sueper, D. T.; Frost, G. J.; Donnelly, S. G.; Schauffler, S.; Atlas, E. L.; Kuster, W. C.; Goldan, P. D.; Hübler, G.; Meagher, J. F.; Fehsenfeld, F. C. Observations of Ozone Formation in Power Plant Plumes and Implications for Ozone Control Strategies. *Science*. **2001**, *292*, 719-723. https://doi.org/10.1126/science.1058113.

(2) Krol, M.; van Stratum, B.; Anglou, I.; Boersma, K. F. Evaluating NOx Stack Plume Emissions Using a High-Resolution Atmospheric Chemistry Model and Satellite-Derived NO2 Columns. *Atmos. Chem. Phys.* **2024**, *24* (14), 8243–8262. https://doi.org/10.5194/acp-24-8243-2024.

(3) Li, Q.; Gabay, M.; Rubin, Y.; Fredj, E.; Tas, E. Measurement-Based Investigation of Ozone Deposition to Vegetation under the Effects of Coastal and Photochemical Air Pollution in the Eastern Mediterranean. *Sci. Total Environ.* **2018**, *645*, 1579–1597. https://doi.org/10.1016/j.scitotenv.2018.07.037.

(4) Dommen, J.; Prévôt, A. S. H.; Neininger, B.; Bäumle, M. Characterization of the Photooxidant Formation in the Metropolitan Area of Milan from Aircraft Measurements. *J. Geophys. Res.: Atmos* **2002**, *107* (22), 8197. https://doi.org/10.1029/2000JD000283.

(5) Corsmeier I, U.; Kalthoff, N.; Vogel, B.; Hammer, M.-U.; Fiedler, F.; Kottmeier, C. H.; Volz-Thomas, A.; Konrad, S.; Glaser, K.; Neininger, B.; Lehning, M.; Jaeschke, W.; Memmesheimer, M.; Rappenglück, B.; Jakobi, G. Ozone and PAN Formation Inside and Outside of the Berlin Plume—Process Analysis and Numerical Process Simulation. *J. Atmos. Chem.* **2002**, *42*, 289–321. https://doi.org/10.1023/A:1015732532499.

(6) Billesbach, D. P. Estimating Uncertainties in Individual Eddy Covariance Flux Measurements: A Comparison of Methods and a Proposed New Method. *Agric. For. Meteorol.* **2011**, *151* (3), 394–405. https://doi.org/10.1016/j.agrformet.2010.12.001.
